# Supplementary material for: Comparison of the CHU-9D and the EQ-5D-Y instruments in children and young people with cerebral palsy: a cross-sectional study
Source: BMJ Open. 2020 Sep 10;10(9):e037089. doi: 10.1136/bmjopen-2020-037089 (PMC7485239; doi:10.1136/bmjopen-2020-037089)
Supplement: Supplementary data [file bmjopen-2020-037089supp001.pdf]

Supplemental Table 1. Linear regression analysis examining association between EQ-5D-Y utility values (dependent variable) and GMFCS level.

	Coefficient	Bootstrap SE	95% CI[a]	R^2	p value
GMFCS level I	reference	-	-	0.231	0.016
GMFCS level II	-0.07	0.06	-0.20, 0.05		
GMFCS level III	-0.43	0.15	-0.73, -0.14		

CI: confidence interval; GMFCS: gross motor function classification system; SE: standard error

[a]bias-corrected and accelerated confidence interval

Supplemental Table 2. Linear regression analysis examining association between CHU-9D utility values (dependent variable) and GMFCS level.

CI: confidence interval; GMFCS: gross motor function classification system; SE: standard

	Coefficient	Bootstrap SE	95% CI[a]	R^2	p value
GMFCS level I	reference	-	-	0.071	0.170
GMFCS level II	0.02	0.03	-0.03, 0.07		
GMFCS level III	-0.07	0.05	-0.17, 0.02		

error

[a]bias-corrected and accelerated confidence interval
