# Supplementary material for: Comparison of the CHU-9D and the EQ-5D-Y instruments in children and young people with cerebral palsy: a cross-sectional study
Source: BMJ Open. 2020 Sep 10;10(9):e037089. doi: 10.1136/bmjopen-2020-037089 (PMC7485239; doi:10.1136/bmjopen-2020-037089)
Supplement: Author's manuscript [file bmjopen-2020-037089.draft_revisions.pdf]

BMJ Open is committed to open peer review. As part of this commitment we make the peer review history of every article we publish publicly available.

When an article is published we post the peer reviewers' comments and the authors' responses online. We also post the versions of the paper that were used during peer review. These are the versions that the peer review comments apply to.

The versions of the paper that follow are the versions that were submitted during the peer review process. They are not the versions of record or the final published versions. They should not be cited or distributed as the published version of this manuscript.

BMJ Open is an open access journal and the full, final, typeset and author-corrected version of record of the manuscript is available on our site with no access controls, subscription charges or pay-per-view fees (http://bmjopen.bmj.com).

If you have any questions on BMJ Open's open peer review process please email info.bmjopen@bmj.com

BMJ Open

A comparison of the CHU-9D and the EQ-5D-Y instruments in adolescents with cerebral palsy.

Journal:	*BMJ Open*
Manuscript ID	bmjopen-2020-037089
Article Type:	Original research
Date Submitted by the Author:	17-Jan-2020
Complete List of Authors:	Ryan, Jennifer; Royal College of Surgeons in Ireland, Department of Public Health and Epidemiology; Brunel University, College of Health and Life Sciences<br>McKay, Ellen; Royal College of Surgeons in Ireland, Department of Public Health and Epidemiology<br>Anokye, Nana; Brunel University, Health Economics Research Group<br>Noorkoiv, Marika<br>Theis, Nicola; University of Gloucestershire, School of Sport and Exercise<br>Lavelle, Grace; King's College London, Institute of Psychiatry
Keywords:	HEALTH ECONOMICS, Developmental neurology & neurodisability < PAEDIATRICS, PAEDIATRICS

SCHOLARONE™
Manuscripts

BMJ

I, the Submitting Author has the right to grant and does grant on behalf of all authors of the Work (as defined in the below author licence), an exclusive licence and/or a non-exclusive licence for contributions from authors who are: i) UK Crown employees; ii) where BMJ has agreed a CC-BY licence shall apply, and/or iii) in accordance with the terms applicable for US Federal Government officers or employees acting as part of their official duties; on a worldwide, perpetual, irrevocable, royalty-free basis to BMJ Publishing Group Ltd ("BMJ") its licensees and where the relevant Journal is co-owned by BMJ to the co-owners of the Journal, to publish the Work in this journal and any other BMJ products and to exploit all rights, as set out in our licence.

The Submitting Author accepts and understands that any supply made under these terms is made by BMJ to the Submitting Author unless you are acting as an employee on behalf of your employer or a postgraduate student of an affiliated institution which is paying any applicable article publishing charge ("APC") for Open Access articles. Where the Submitting Author wishes to make the Work available on an Open Access basis (and intends to pay the relevant APC), the terms of reuse of such Open Access shall be governed by a Creative Commons licence – details of these licences and which Creative Commons licence will apply to this Work are set out in our licence referred to above.

Other than as permitted in any relevant BMJ Author's Self Archiving Policies, I confirm this Work has not been accepted for publication elsewhere, is not being considered for publication elsewhere and does not duplicate material already published. I confirm all authors consent to publication of this Work and authorise the granting of this licence.

For peer review only

A comparison of the CHU-9D and the EQ-5D-Y instruments in adolescents with cerebral palsy.

Jennifer M. Ryan[1,2], Ellen McKay[1], Nana Anokye[2], Marika Noorkoiv[2], Nicola Theis[3], Grace Lavelle[4]

[1]Department of Public Health and Epidemiology, RCSI, Ireland

[2]College of Health and Life Sciences, Brunel University London, United Kingdom

[3]School of Sport and Exercise, University of Gloucestershire, Gloucester, United Kingdom

[4]Institute of Psychiatry, Psychology and Neuroscience, King's College London, United Kingdom

Corresponding author: Jennifer M. Ryan, Department of Public Health and Epidemiology, RCSI, Ireland. Email: jenniferryan@rcsi.com

Word count: 3402

For peer review only

Abstract

Objective: To compare the performance of the EuroQol 5D-youth (EQ-5D-Y) and child health utility 9D (CHU-9D) for assessing health-related quality of life (HRQoL) in adolescents with cerebral palsy (CP).

Design: Cross-sectional study

Setting: England

Participants: Sixty-three adolescents with cerebral palsy aged 10-19 years in Gross Motor Function Classification System levels I-III.

Main outcome measures: All participants completed the EQ-5D-Y and CHU-9D. The distribution of responses across levels of the EQ-5D-Y and CHU-9D dimensions and associations between each dimension of the EQ-5D-Y and the CHU-9D were examined. The construct validity of each instrument was assessed by examining associations between responses on each dimension and functional mobility.

Results: Missing data were <5% for both instruments. The EQ-5D-Y exhibited a ceiling effect, with 32% of participants reporting "full" health. Correlations between EQ-5D-Y and CHU-9D dimensions were weak to moderate (r=0.25 to r=0.59, p<0.05). Functional mobility was associated with "mobility" and "looking after myself" dimensions of the EQ-5D-Y (p=0.007 and p=0.036). Functional mobility was not associated with any dimension on the CHU-9D or utility score.

Conclusions: The EQ-5D-Y and CHU-9D are feasible measures of HRQoL in adolescents with CP. However, they data from the two instruments may not be comparable. Given the CHU-9D assesses concepts that influence HRQoL among adolescents with CP and has less of a ceiling effect than the EQ-5D-Y, the CHU-9D may be preferable to use in this population

Keywords: health economics; developmental neurology and neurodisability; paediatrics; cerebral palsy; EQ-5D-Y, CHU-9D

For peer review only - http://bmjopen.bmj.com/site/about/guidelines.xhtml

Strengths and limitations of this study

- This is the first study to compare the performance of the EQ-5D-Y and CHU-9D in a clinical population.
- The findings are limited to ambulatory individuals with cerebral palsy.
- We were unable to compare utilities between the EQ-5D-Y and CHU-9D as there is no set of values for the EQ-5D-Y and use of the adult EQ-5D tariff in children is not recommended

For peer review only

For peer review only - http://bmjopen.bmj.com/site/about/guidelines.xhtml

Cerebral palsy (CP) is a heterogeneous disorder characterised by abnormal movement and posture. It is often co-existent with epilepsy, intellectual disability, and language, communication, or behavioural difficulties [1]. Its prevalence is 2 per 1,000 live births [2]; approximately 110,000 people live with CP in the UK [3]. Cerebral palsy is a lifelong condition. Management of CP encompasses medical, surgical and rehabilitation interventions [1]. While a large volume of research has examined the effectiveness of interventions for people with CP[4] there is limited research examining cost-effectiveness of such interventions.

Economic evaluation is used to inform the efficient allocation of resources in a healthcare setting. The cost utility analysis (CUA) is the type of economic evaluation recommended by the National Institute for Health and Care Excellence (NICE) particularly for interventions funded by NHS and personal social services [5]. The CUA commonly describes the relationship between costs and health benefits as the cost per quality-adjusted life year (QALY). QALYs incorporate both quantity and quality of life. QALYs are commonly calculated using an assessment of health-related quality of life (HRQoL) obtained from a preference-based instrument. Such instruments can provide a health state utility value, where 0 indicates a health state of equivalent value to being dead and 1.0 indicates full health, by applying a pre-specified algorithm based on preferences for health status identified in a specific population [6].

Generic HRQoL instruments are recommended for use in economic evaluations as they allow comparison across health care interventions and populations. However, if scores from generic measures differ, estimates of cost-effectiveness will be impacted, leading to uncertain conclusions regarding whether an intervention provides value for money. A number of generic measures have been used to obtain health state utility values from paediatric populations [7]. The most commonly used are the EuroQol 5D (EQ-5D), the Child Health Utility 9D (CHU-9D), the EuroQol 5D Youth version (EQ-5D-Y) and the Health Utilities Index 2 and 3 (HUI-2 and HUI-3) [7]. To date, however, only the HUI-2 and HUI-3 have been used to elicit utility values from young people with CP and significant variation was reported in utility values [8].

The CHU-9D and EQ-5D-Y are two generic preference-based HRQoL instruments that were designed specifically for young people [7]. The EQ-5D-Y is a youth modified version of the adult instrument, the EQ-5D, which was developed by revising the content and wording of the adult instrument [9]. The CHU-9D was developed from the outset for young people based on in-depth interviews with young people with chronic and acute health conditions [10]. Although the CHU-9D and EQ-5D-Y

For peer review only - http://bmjopen.bmj.com/site/about/guidelines.xhtml

appear to be appropriate for assessing HRQoL in an adolescent population [11], their performance has not been examined among young people with CP.

Given the financial cost of CP per annum is approximately AU$1.5 billion [12] and a wide range of interventions are currently available for people with CP [4], there is an increasing need for economic evaluation in this area. Prior to conducting economic evaluations, the performance of the CHU-9D and EQ-5D-Y in young people with CP requires evaluation. The aim of this study was to compare the performance of the EQ-5D-Y and CHU-9D for assessing HRQoL in a community-based sample of young people with CP.

Methods

Sample

Adolescents with CP who participated in a randomised controlled trial examining the effects of progressive resistance training were included in this study. Data collected at baseline were used for this cross-sectional study. Participants were recruited from eight National Health Service (NHS) trusts in England, a special education needs school, a University and a primary care organisation in London, national organisations for people with disabilities, and by word of mouth. Adolescents aged 10-19 years with spastic CP and the ability to walk independently with or without a mobility aid (i.e., Gross Motor Function Classification System [GMFCS] levels I-III) were included in the study. Exclusion criteria were orthopaedic surgery of the lower limbs in the past 12 months, botulinum toxin type A injections or serial casting in the past 6 months, and insufficient cognition to comply with assessment procedures and the training programme. The study was approved by Brunel University London's College of Health and Life Sciences Research Ethics Committee and the Surrey Borders Research Ethics Committee (ref: 15/LO/0843). Participants 16 years and older provided written consent. Those under 16 years provided assent alongside written consent from a parent or guardian.

For peer review only

Data on the person's demographics, condition and HRQoL were collected using standardised questionnaires during an interview with a researcher at one time-point. Anatomical distribution was described as unilateral or bilateral [13]. Functional mobility was classified according to the GMFCS. The GMFCS is a five level classification system, where level I indicates most able and level V indicates most limited. Those in GMFCS level I are able to walk and run and climb stairs without assistance. Those in level II are able to walk in most settings but may use a hand-held mobility device indoors or wheeled mobility to travel long distances. Those in level III can walk using a hand-held mobility device but use a wheelchair or powered mobility outdoors. Participants selected a statement that

For peer review only - http://bmjopen.bmj.com/site/about/guidelines.xhtml

best described their mobility based on descriptors of each GMFCS level.[14] Two physiotherapists retrospectively cross-referenced subjective ratings of GMFCS level against video recordings of participants, obtained as part of the baseline assessment.

Utility measurement

The EQ-5D-Y assesses a person's health across five dimensions. The five dimensions are "mobility", "looking after myself", "doing usual activities", "having pain or discomfort" and "feeling worried", "sad or unhappy" [9]. Each dimension is rated on one of three levels (no problems, some problems, and a lot of problems) that describes a person's health today [9]. The EQ-5D-Y was developed by reviewing the applicability of the EQ-5D domain concepts and wording for children and adolescents [9]. The EQ-5D-Y is suitable for use in young people aged 8-19 years [15]. However, there is no set of values for the EQ-5D-Y. Although the EQ-5D-Y has been used with the adult EQ-5D tariff, it is not recommended [16]. We therefore did not calculate utilities for the EQ-5D-Y.

There are 9 dimension in the CHU-9D: "worried", "sad", "pain", "tired", "annoyed", "schoolwork", "sleep", "daily routine" and "ability to join in activities" [10]. For each dimension, the person describes how they are today according to one of five levels based on severity (e.g., not worried, a little bit worried, a bit worried, quite worried, very worried), which were determined from in-depth interviews with young people [10]. The CHU-9D is suitable for use in young people aged 7-17 years [10, 17]. The scoring algorithm based on the preferences of the UK adult general population was used to estimate utilities [18]. This method has been shown to be appropriate to use for young people [16].

Patient and Public Involvement

Young people with and without CP were involved in the design, conduct and dissemination plans of our research relating to the randomised controlled trial examining the effects of progressive resistance training.

Statistical analysis

Distribution of continuous variables was examined using histograms. Mean and standard deviation [SD] were reported for data that were normally distributed. Median and interquartile range were reported for data that were not normally distributed. For categorical data, frequency and percentage were reported. To compare the performance of the CHU-9D and the EQ-5D-Y, we firstly examined associations between each dimension of the EQ-5D-Y and CHU-9D by calculating

For peer review only - http://bmjopen.bmj.com/site/about/guidelines.xhtml

Spearman's correlation coefficients. We also examined the association between CHU-9D utility score and levels of each EQ-5D-Y domain by conducting linear regression analysis using a bootstrap procedure. The construct validity of each instrument was examined by comparing the number of people experiencing no problems versus any problems for each CHU-9D and EQ-5D-Y dimension across levels of functional mobility, defined by the GMFCS, using a Chi^2 test. We also conducted linear regression analysis using a bootstrap procedure to compare CHU-9D utility score across GMFCS levels. Bias corrected and accelerated bootstrap confidence intervals (CIs) were calculated from 2,000 replicates [19]. All statistical analyses were executed using Stata version 13.

Results

Sixty-four participants were recruited to the study. One person did not complete the EQ-5D-Y or CHU-9D. Therefore, 63 participants were included in the analysis. Table 1 describes participant characteristics. The mean±SD age was 13.7±2.5 yr. The majority of participants (86%) were in GMFCS levels I or II indicating a mild lower limb impairment. The majority of participants were White British (59%) and attended a mainstream school (71%).

The percentages of reported problems across dimensions of the EQ-5D-Y and CHU-9D, respectively, are presented in Tables 2 and 3. For the CHU-9D, two participants (3.2%) did not provide a response to the schoolwork dimension and one participant (1.6%) did not provide a response to the ability to "join in activities" dimension. For the EQ-5D-Y, one participant (1.6%) did not provide a response to the "looking after myself" dimension.

For the EQ-5D-Y and CHU-9D, respectively, 20 participants (31.8%) and 11 participants (17.5%) reported full health (i.e., they reported the least severe level for all dimensions). For all dimensions of the EQ-5D-Y, the majority of respondents reported themselves in the least severe level (i.e. no problems). However, responses for most dimensions of the EQ-5D-Y were spread across all levels, from no problems to a lot of problems. The exception to this was the "worried, sad or unhappy" dimension; no participant reported feeling very worried, sad or unhappy. Similarly, for all dimensions of the CHU-9D except for "tired", the majority of participants reported themselves in the least severe level. However, responses were not spread across all levels for each dimension. For the "worried", "sad", "pain", "annoyed", " "sleep" and "daily routine" dimensions of the CHU-9D, no participant reported the most severe level.

For peer review only - http://bmjopen.bmj.com/site/about/guidelines.xhtml

For peer review only

The correlations between EQ-5D-Y and CHU-9D dimensions are presented in Table 4. Generally, correlations were weak to moderate. The highest correlation was found between “daily routine” on the CHU-9D and “looking after myself” on the EQ-5D-Y (r=0.59, p<0.001). The next highest correlations were found for “schoolwork” on the CHU-9D and “mobility” and “pain or discomfort” on the EQ-5D-Y (r = 0.56, p<0.001, and r=0.55, p<0.001, respectively). Moderate correlations were also found for similar dimensions such as “pain” on the CHU-9D and “pain or discomfort” on the EQ-5D-Y (r=0.47, p<0.001) and “able to join in activities” on the CHU-9D and “activities” on the EQ-5D-Y (r=0.54, p<0.001).

Not all dimensions that may be considered similar had high correlations. For example, “worried” on the CHU-9D and “worried, sad or unhappy” on the EQ-5D-Y were only weakly correlated (r=0.25, p=0.048). However, “sad” on the CHU-9D was more strongly correlated with “worried, sad or unhappy” on the EQ-5D-Y (r=0.43, p<0.001). Although “daily routine” on the CHU-9D was not associated with “mobility” on the EQ-5D-Y, “able to join in activities” was associated with “mobility” (r=0.49, p<0.001). “Worried” and “schoolwork” on the CHU-9D were associated with all dimensions of the EQ-5D-Y. In contrast, “sad” on the CHU-9D was only associated with one EQ-5D-Y dimension.

The median±IQR utility of the CHU-9D was 0.92±0.13 (range 0.56 to 1.00; n=61). The median±IQR utility of the CHU-9D according to EQ-5D-Y dimensions is presented in Table 2. For all dimensions of the EQ-5D-Y, the median CHU-9D utility score decreased with increasing levels of severity on the EQ-5D-Y (p<0.001 for all). Moderate correlations were observed between level of severity on EQ-5D-Y dimensions and CHU-9D utility score (R^2 = 0.188 to R^2 = 0.363; Table 2).

As presented in Table 5, the percentage of people reporting problems on the “mobility” and “looking after myself” dimensions of the EQ-5D-Y differed according to GMFCS level (p=0.007 and p=0.036, respectively), with percentage of problems decreasing across GMFCS levels I to III. The percentage of people reporting problems was not associated with GMFCS level for any other EQ-5D-Y dimension. There was also no evidence of an association between level of severity on any dimension of the CHU-9D and GMFCS level (Table 6). Median±IQR CHU-9D utility score was 0.92±0.14 for participants in GMFCS level I (range 0.56 to 1.00; n=29), 0.92±0.11 for participants in GMFCS level II (range 0.76 to 1.00; n=25), and 0.83±0.17 for participants in GMFCS level III (range 0.65 to 1.00; n=7). There was no difference in CHU-9D utility score across GMFCS levels (p=0.170).

Discussion

For peer review only - http://bmjopen.bmj.com/site/about/guidelines.xhtml

The aim of this study was to compare the performance of the EQ-5D-Y and CHU-9D for assessing HRQoL in young people with CP. To our knowledge, this is the first study to compare the two measures in a clinical population. There was little missing data suggesting that the two instruments are feasible to use when administered by interview in this population. Correlations between individual dimensions on each instrument were weak to moderate, highlighting that ratings on dimensions that may seem to measure similar concepts are not identical. However, there was some evidence of convergent validity between the two instruments as the CHU-9D utility score decreased across levels of all EQ-5D-Y dimensions. Ratings for “mobility” and “looking after myself” dimensions on the EQ-5D-Y differed according to functional mobility as assessed by the GMFCS. However, ratings on other dimensions of the EQ-5D-Y, all dimensions of the CHU-9D and the CHU-9D utility score did not differ between those in GMFCS level I, II and III.

To date, the performance of only two generic measures, the HUI-3 and the Assessment of Quality of Life (AQoL), has been examined among children with CP [8]. The HUI-3 and AQoL were strongly correlated in adolescents with CP in GMFCS levels I-V [20]. The HUI-3 was only weakly correlated, however, with a condition-specific measure of HRQoL, the Quality of Life Instrument for People with Developmental Disabilities [21]. This weak correlation highlights that condition-specific measures and generic measures of HRQoL may not be assessing similar concepts. However, unlike generic measures, condition-specific measures are not recommended for evaluations across different conditions [22]. Unlike the current study, both the HUI-3 and AQoL demonstrated moderate-to-high correlations with GMFCS level [20, 21]. However, correlations were assessed across five GMFCS levels, as opposed to three in the current study.

The median CHU-9D utility score for each GMFCS level was not comparable to the mean utility score obtained from the HUI-3 and AQoL in previous studies [20, 21]. Rosenbaum reported a mean utility of 0.84, 0.50 and 0.39 for adolescents in GMFCS levels I, II and III, respectively from the HUI-3 [21]. Young reported a mean utility of 0.67, 0.59 and 0.43 from the HUI-3 and 0.58, 0.53 and 0.31 from the AQoL for adolescents in GMFCS levels I, II and III, respectively [20]. These were much lower than the median utility from the CHU-9D of 0.92, 0.92 and 0.83 for adolescents in GMFCS levels I, II and III. Discrepancies may be due to differences between samples or due to differences in the algorithms used to derive utility values. One study applied preferences from the general adult population in Canada [21] and the second study did not state the preferences used to derive utility values [20].

For peer review only - http://bmjopen.bmj.com/site/about/guidelines.xhtml

Two studies have compared the EQ-5D-Y and CHU-9D in children and adolescents with typical development [11, 16]. Similar to this study, a ceiling effect was reported for the EQ-5D-Y for Australian adolescents; 40% of respondents were classified at full health [11]. In the current study, almost twice as many participants reported full health when using the EQ-5D-Y compared to the CHU-9D. This suggests that the CHU-9D is more sensitive to varying severities of health potentially because of the larger number of levels for each dimension and because the levels were determined from in-depth interviews with young people [10].

The median CHU-9D utility score among adolescents in this study was higher than that reported by adolescents with typical development (0.92 versus 0.83) [11]. The median CHU-9D utility observed among adolescents with CP was more similar to the median observed among children aged 6-7 years with typical development [16]. This may be because the study of children aged 6-7 years used the same algorithm to estimate utilities as the current study, while the study of adolescents used an algorithm developed in the Australian population. However, it is also possible that adolescents with CP have better HRQoL than adolescents with typical development. A study of a large sample of adolescents with CP across Europe found that adolescents with CP had better QoL in five domains (moods and emotions, self-perception, autonomy, relationships with parents, school life) compared to adolescents in the general population matched for age, sex and country [23]. QoL was worse among adolescents with CP in the social support domain only [23].

We found weak to moderate correlations between dimensions on the EQ-5D-Y and CHU-9D, despite both instruments being administered at the same time-point, by the same researcher and in the same environment. This finding has important clinical implications for choosing an instrument to assess HRQoL in adolescents with CP and interpreting results from the CHU-9D and EQ-5D-Y. In a study of Australian adolescents with typical development, correlations between the two instruments were stronger [11]. The strongest correlation between EQ-5D-Y and CHU-9D dimensions was between pain/discomfort and pain (r=0.753) [11]. However, pain/discomfort and pain were only moderately correlated in adolescents with CP (r=0.47). Many children and adolescents with CP experience frequent pain [24], and as a result, their interpretation of the EQ-5D-Y question about pain and discomfort may differ to their interpretation of the CHU-9D question about pain only. Indeed, fifty-six percent of adolescents with CP reported no pain or discomfort on the EQ-5D-Y, while 62% reported no pain on the CHU-9D. The instruments also both asked participants to rate their pain today, rather than over a period of time, which may explain this finding.

For peer review only - http://bmjopen.bmj.com/site/about/guidelines.xhtml

The strength of a number of correlations between EQ-5D-Y and CHU-9D dimensions differed among adolescents with CP compared to adolescents with typical development [11]. The correlation between “worried, sad and unhappy” on the EQ-5D-Y and “worried” on the CHU-9D was 0.25 in adolescents with CP but was one of the strongest correlations found among adolescents with typical development (r=0.697). In contrast, “schoolwork” on the CHU-9D was very weakly correlated with EQ-5D-Y dimensions among adolescents with typical development [11]. Whereas the correlations between “schoolwork” and “mobility” and “schoolwork” and “pain” were two of the strongest correlations observed among adolescents with CP. “Able to join in activities” and “mobility”, and “daily routine” and “looking after myself” were moderately correlated in adolescents with CP but only weakly correlated in adolescents and children with typical development [11, 16].

The CHU-9D is the only generic measure that was specifically designed for children and adolescents. The EQ-5D-Y is a modified version of the most widely used generic measure in adults, the EQ-5D. The performance of these measures has not been examined in people with CP to date. This study suggests that the CHU-9D may be more likely than the EQ-5D-Y to capture dimensions of HRQoL that are important to people with CP. The CHU-9D includes more questions about psychological difficulties and pain, which are associated with QoL among adolescents with CP [23]. Although mobility is not a dimension on the CHU-9D, correlations between “mobility” on the EQ-5D-Y and CHU-9D dimensions of “worried”, “pain”, “sad”, “tired”, “schoolwork”, “sleep” and “activities”, suggests that the impact of mobility on HRQoL may be captured by these factors rather than mobility alone. Indeed, this study did not find that functional mobility, as assessed by the GMFCS, was associated with overall HRQoL or several dimensions of HRQoL. Similarly, a large European study found that only the autonomy subscale of QoL differed across GMFCS levels I-III among adolescents with CP [23].

Limitations

The sample included adolescents with CP in GMFCS levels I-III only, which represents about 70% of individuals with CP [25]. The findings may not be applicable to non-ambulatory adolescents. As the sample volunteered to participate, they may have a higher QoL than those who did not volunteer. We were unable to compare utilities between the EQ-5D-Y and CHU-9D as there is no set of values for the EQ-5D-Y and use of the adult EQ-5D tariff in children is not recommended [16].

In conclusion, the findings of this study illustrate the potential for the CHU-9D and the EQ-5D-Y to be used among adolescents with CP. However, they do not appear to measure the same constructs in

For peer review only - http://bmjopen.bmj.com/site/about/guidelines.xhtml

adolescents with CP and data from the two instruments may not be comparable. Given the CHU-9D assesses concepts that influence HRQoL among adolescents with CP and has less of a ceiling effect than the EQ-5D-Y, the CHU-9D may be preferable to use in this population. Additionally, child and adolescents preferences are not currently available for the EQ-5D-Y and is therefore not recommended for eliciting utility values. The CHU-9D provides a generic measure that may be used to compare HRQoL across populations including adolescents with CP.

Competing interests: None declared.

Author statement: JR and GL conceived the study. JR and EM performed the analysis and drafted the manuscript. All authors have made substantial revisions to earlier drafts and approved the final manuscript.

Data sharing statement: Data are available upon reasonable request.

Ethics approval: The study was approved by Brunel University London's College of Health and Life Sciences Research Ethics Committee and the Surrey Borders Research Ethics Committee (ref: 15/LO/0843).

Funding: Action Medical Research and the Chartered Society of Physiotherapy Charitable Trust have jointly funded this project, and it is supported by a generous grant from The Henry Smith Charity (GN2340).

References

1. Colver, A., Fairhurst, C., & Pharoah, P.O. (2014) Cerebral palsy. *Lancet,* 383(9924), 1240-1249.
2. Oskoui, M., Gazzellone, M.J., Thiruvahindrapuram, B., Zarrei, M., Andersen, J., Wei, J., Wang, Z., Wintle, R.F., Marshall, C.R., Cohn, R.D., Weksberg, R., Stavropoulos, D.J., Fehlings, D., Shevell, M.I., Scherer, S.W. (2015) Clinically relevant copy number variations detected in cerebral palsy. *Nature Communications*, 7949.
3. DH Long-term Conditions NSF Team. (2005) The National Service Framework for Long-Term Conditions. *Department of Health*.
4. Novak, I., McIntyre, S., Morgan, C., Campbell, L., Dark, L., Morton, N., Stumbles, E., Wilson, S.A., Goldsmith, S. (2013) A systematic review of interventions for children with cerebral palsy: state of the evidence. *Developmental Medicine And Child Neurology,* 55(10), 885-910.
5. NICE. Developing NICE guidelines: the manual London; 2018.
6. Whitehead, S.J. & Ali, S. (2010) Health outcomes in economic evaluation: the QALY and utilities. *British Medical Bulletin,* 96, 5-21.
7. Thorrington, D. & Eames, K. (2015) Measuring Health Utilities in Children and Adolescents: A Systematic Review of the Literature. *PloS One,* 10(8), e0135672.

For peer review only - http://bmjopen.bmj.com/site/about/guidelines.xhtml

8. Tonmukayakul, U., Le, L.K., Mudiyanselage, S.B., Engel, L., Bucholc, J., Mulhern, B., Carter, R., & Mihalpoulos, C. (2019) A systematic review of utility values in children with cerebral palsy. *Quality of Life Research*, 28(1), 1-12.
9. Wille, N., Badia, X., Bonsel, G., Burstrom, K., Cavrini, G., Devlin, N., Egmar, A.C., Greiner, W., Gusi, N., Herdman, M., Jelsma, J., Kind, P., Scalone, L., & Ravens-Sieberer, U. (2010) Development of the EQ-5D-Y: a child-friendly version of the EQ-5D. *Quality of Life Research*, 19(6), 875-886.
10. Stevens K. (2009) Developing a descriptive system for a new preference-based measure of health-related quality of life for children. *Quality of Life Research*, 18(8), 1105-1113.
11. Chen, G., Flynn, T., Stevens, K., Brazier, J., Huynh, E., Sawyer, M., Roberts, R., & Ratcliffe, J. (2015) Assessing the Health-Related Quality of Life of Australian Adolescents: An Empirical Comparison of the Child Health Utility 9D and EQ-5D-Y Instruments. *Value in Health,* 18(4), 432-438.
12. Access Economics (2008) The economic impact of cerebral palsy in Australia in 2007. Canberra, Australia: Access Economics.
13. Rosenbaum, P., Paneth, N., Leviton, A., Goldstein, M., Bax, M., Damiano, D., Dan, B., & Jacobsson, B. (2007) A report: the definition and classification of cerebral palsy April 2006. *Developmental Medicine And Child Neurology Supplement*, 109, 8-14.
14. Palisano, R., Rosenbaum, P., Walter, S., Russell, D., Wood, E., Galuppi, B. (1997) Development and reliability of a system to classify gross motor function in children with cerebral palsy. *Developmental Medicine And Child Neurology*, 39(4), 214-223.
15. Ravens-Sieberer, U., Wille, N., Badia, X., Bonsel, G., Burstrom, K., Cavrini, G., Devlin, N., Egmar, A.C., Gusi, N., Herdman, M., Jelsma, J., Kind, P., Olivares, P.R., Scalone, L., & Greiner, W. (2010) Feasibility, reliability, and validity of the EQ-5D-Y: results from a multinational study. *Quality Of Life Research,* 19(6), 887-897.
16. Canaway, A.G. & Frew, E.J. (2013) Measuring preference-based quality of life in children aged 6-7 years: a comparison of the performance of the CHU-9D and EQ-5D-Y--the WAVES pilot study. *Quality Of Life Research,* 22(1), 173-183.
17. Ratcliffe, J., Stevens, K., Flynn, T., Brazier, J., & Sawyer, M. (2012) An assessment of the construct validity of the CHU9D in the Australian adolescent general population. *Quality Of Life Research, 21*(4), 717-725.
18. Stevens, K. (2012) Valuation of the Child Health Utility 9D Index. *Pharmacoeconomics,* 30(8), 729-747.
19. Good P. (2005) Permutation, Parametric, and Bootstrap Tests of Hypotheses. Third edition. Springer.
20. Young, N.L., Rochon, T.G., McCormick, A., Law, M., Wedge, J.H., & Fehlings, D. (2010) The health and quality of life outcomes among youth and young adults with cerebral palsy. *Archives Of Physical Medicine And Rehabilitation,* 91(1), 143-148.
21. Rosenbaum, P.L., Livingston, M.H., Palisano, R.J., Galuppi, B.E., Russell, D.J. (2007) Quality of life and health-related quality of life of adolescents with cerebral palsy. *Developmental Medicine And Child Neurology,* 49(7), 516-521.
22. Seow, L.S.E., Tan, T.H.G., Abdin, E., Chong, S.A., & Subramaniam, M. (2019) Comparing disease-specific and generic quality of life measures in patients with schizophrenia. *Psychiatry Research,* 273, 387-393.
23. Colver, A., Rapp, M., Eisemann, N., Ehlinger, V., Thyen, U., Dickinson, H.O., Parkes, J., Parkinson, K., Nystrand, M., Fauconnier, J., Marcelli, M., Michelsen, S.I., Arnaud, C. (2015) Self-reported quality of life of adolescents with cerebral palsy: a cross-sectional and longitudinal analysis. *Lancet,* 385(9969), 705-716.
24. Westbom, L., Rimstedt, A., & Nordmark, E. (2017) Assessments of pain in children and adolescents with cerebral palsy: a retrospective population-based registry study. *Developmental Medicine And Child Neurology,* 59(8), 858-863.

For peer review only - http://bmjopen.bmj.com/site/about/guidelines.xhtml

25. Children and young people with cerebral palsy in Northern Ireland (1981-2008). A comprehensive report from the Northern Ireland Cerebral Palsy Register. HSC Public Health Agency and Queen's University Belfast.

For peer review only

For peer review only - http://bmjopen.bmj.com/site/about/guidelines.xhtml

Table 1. Participant characteristics

	n (%)	Mean (SD)	Range
Age, yr	63	13.7 (2.5)	10 to 19
Female	26 (41.3)		
Height, cm	63	154.3 (12.7)	131.5 to 180.9
Mass, kg	63	49.3 (13.7)	27.4 to 78.5
**Ethnicity**			
White British	37 (58.7)		
Black or Black British	5 (7.9)		
Asian or Asian British	9 (14.3)		
Other	12 (19.0)		
**GMFCS level**			
I	29 (46.0)		
II	25 (39.7)		
III	9 (14.3)		
**Distribution**			
Unilateral	31 (49.2)		
Bilateral	32 (50.8)		
**Type of School**			
Attends mainstream school	45 (71.4)		
Attends SEN	13 (20.6)		
Attends further college education	2 (3.2)		
Attends University	3 (4.8)		

SD: standard deviation; GMFM: Gross motor function classification system; SEN: Special Needs Education

For peer review only

For peer review only - http://bmjopen.bmj.com/site/about/guidelines.xhtml

Table 2. Number and percentage of participants reporting problems across CHU-9D dimensions

EQ-5D-Y dimensions	levels	Frequency (%)	n	Median±IQR CHU-9D utility score	R^2	p value
Mobility	No	50.8	32	0.95±0.09	0.281	<0.001
	Some	44.4	28	0.84±0.13		
	A lot of	4.8	3	0.75±0.05		
Looking after myself	No	66.7	42	0.94±0.09	0.208	<0.001
	Some	27.0	17	0.83±0.10		
	A lot of	4.8	3	0.72±0.27		
	Missing	1.6	1	0.78		
Usual activities	No	66.7	42	0.93±0.11	0.359	<0.001
	Some	28.6	18	0.83±0.15		
	A lot of	4.8	3	0.72±0.16		
Pain or discomfort	No	55.6	35	0.95±0.11	0.363	<0.001
	Some	38.1	24	0.86±0.16		
	A lot of	6.3	4	0.72±0.24		
Worried, sad or unhappy	Not	84.1	53	0.92±0.11	0.188	<0.001
	A bit	15.9	10	0.79±0.05		
	Very	0	0	-		

IQR: interquartile range

For peer review only

For peer review only - http://bmjopen.bmj.com/site/about/guidelines.xhtml

Table 3. Number and percentage of participants reporting problems across CHU-9D dimensions

CHU-9D dimensions	levels	Frequency (%)	n
Worried	Not worried	82.5	52
	A little bit	15.9	10
	A bit	1.6	1
	Quite	0	0
	Very	0	0
Sad	Not sad	93.7	59
	A little bit	3.2	2
	A bit	3.2	2
	Quite	0	0
	Very	0	0
Pain	No pain	61.9	39
	A little bit	30.2	19
	A bit	3.2	2
	Quite	4.8	3
	A lot	0	0
Tired	Not tired	36.5	23
	A little bit	42.9	27
	A bit	9.5	6
	Quite	7.9	5
	Very	3.2	2
Annoyed	Not annoyed	84.1	53
	A little bit	11.1	7
	A bit	3.2	2
	Quite	1.6	1
	Very	0	0
Schoolwork/homework	No problems	63.5	40
	Few problems	17.5	11
	Some problems	14.3	9
	Many problems	0	0

For peer review only

	Can't do schoolwork	1.6	1
	Missing	3.2	2
Sleep	No problems	71.4	45
	Few problems	20.6	13
	Some problems	4.8	3
	Many problems	3.2	2
	Can't sleep	0	0
Daily routine	No problems	74.6	47
	Few problems	15.9	10
	Some problems	7.9	5
	Many problems	1.6	1
	Can't do daily routine	0	0
Able to join in activities	Any activities	57.1	36
	Most activities	27.0	17
	Some activities	7.9	5
	Few activities	4.8	3
	No activities	1.6	1
	Missing	1.6	1

For peer review only

For peer review only - http://bmjopen.bmj.com/site/about/guidelines.xhtml

Table 4. Spearman rank correlation coefficients between CHU-9D and EQ-5D-Y dimensions

**EQ-5D-Y dimensions**		**CHU-9D dimensions**								
		**Worried**	**Sad**	**Pain**	**Tired**	**Annoyed**	**Schoolwork**	**Sleep**	**Daily routine**	**Activities**
**Mobility**	*r*	***0.30***	*-0.01*	***0.35***	***0.36***	*0.19*	***0.56***	***0.31***	*0.19*	***0.49***
	p	**0.017**	0.950	**0.006**	**0.004**	0.140	**<0.001**	**0.013**	0.144	**<0.001**
	n	**63**	63	**63**	**63**	63	**61**	**63**	63	**62**
**Looking after myself**	*r*	***0.26***	*-0.04*	*0.21*	*0.22*	*0.08*	***0.39***	***0.27***	***0.59***	***0.36***
	p	**0.040**	0.741	0.098	0.086	0.525	**0.002**	**0.031**	**<0.001**	**0.004**
	n	**62**	62	62	62	62	**60**	**62**	**62**	**61**
**Activities**	*r*	***0.36***	*0.08*	***0.30***	*0.23*	***0.25***	***0.44***	***0.38***	***0.35***	***0.54***
	p	**0.004**	0.543	**0.017**	0.065	**0.049**	**<0.001**	**0.002**	**0.006**	**<0.001**
	n	**63**	63	**63**	63	**63**	**61**	**63**	**63**	**62**
**Pain or discomfort**	*r*	***0.33***	*0.07*	***0.47***	***0.40***	*0.22*	***0.55***	***0.39***	***0.25***	***0.32***
	p	**0.008**	0.600	**<0.001**	**0.001**	0.090	**<0.001**	**0.002**	**0.048**	**0.011**
	n	**63**	63	**63**	**63**	63	**61**	**63**	**63**	**62**
**Worried, sad, unhappy**	*r*	***0.25***	***0.43***	*0.19*	***0.26***	***0.53***	***0.39***	*-0.01*	***0.33***	*0.15*
	p	**0.048**	**<0.001**	0.143	**0.039**	**<0.001**	**0.002**	0.944	**0.008**	0.239
	n	**63**	**63**	63	**63**	**63**	**61**	63	**63**	62

Note: correlations with p value <0.05 highlighted in bold

For peer review only - http://bmjopen.bmj.com/site/about/guidelines.xhtml

Table 5. Percentage reporting problems on each EQ-5D-Y dimension across GMFCS level

EQ-5D-Y dimensions	levels	GMFCS level I	GMFCS level II	GMFCS level III	*p value*[a]
		n=29	n=25	n=9	
Mobility	No	69%	44%	11%	0.007
	Some	31%	56%	56%	
	A lot of	0%	0%	33%	
Looking after myself	No	83%	56%	50%	0.036
	Some	17%	40%	25%	
	A lot of	0%	4%	25%	
Usual activities	No	72%	72%	33%	0.072
	Some	28%	24%	44%	
	A lot of	0%	4%	22%	
Pain or discomfort	No	59%	60%	33%	0.348
	Some	38%	36%	44%	
	A lot of	3%	4%	22%	
Worried, sad or unhappy	No	79%	92%	78%	0.380
	Some	21%	8%	22%	
	A lot of	0%	0%	0%	

[a]Comparing number of people experiencing “no” problems versus any problems (i.e., some and a lot of) across GMFCS levels

For peer review only

Table 6. Percentage reporting problems on each CHU-9D dimension across GMFCS level

CHU-9D dimensions	levels	GMFCS level I	GMFCS level II	GMFCS level III	*p value*[a]
		n=29	n=25	n=9	
Worried	Not worried	83%	84%	78%	0.914
	A little bit	17%	16%	11%	
	A bit	0%	0%	11%	
	Quite	0%	0%	0%	
	Very	0%	0%	0%	
Sad	Not sad	86%	100%	100%	0.082
	A little bit	7%	0%	0%	
	A bit	7%	0%	0%	
	Quite	0%	0%	0%	
	Very	0%	0%	0%	
Pain	No pain	55%	72%	56%	0.408
	A little bit	38%	24%	22%	
	A bit	0%	4%	11%	
	Quite	7%	0%	11%	
	A lot	0%	0%	0%	
Tired	Not tired	31%	44%	33%	0.601
	A little bit	52%	32%	44%	
	A bit	7%	12%	11%	
	Quite	10%	8%	0%	

For peer review only - http://bmjopen.bmj.com/site/about/guidelines.xhtml

For peer review only

	Very	0%	4%	11%	
Annoyed	Not annoyed	72%	96%	89%	0.056
	A little bit	17%	4%	11%	
	A bit	7%	0%	0%	
	Quite	4%	0%	0%	
	Very	0%	0%	0%	
Schoolwork/homework	No problems	72%	64%	43%	0.104
	Few problems	17%	16%	29%	
	Some problems	10%	20%	14%	
	Many problems	0%	0%	14%	
	Can't do schoolwork	0%	0%	0%	
Sleep	Not	79%	68%	56%	0.343
	A little bit	10%	28%	33%	
	A bit	7%	4%	0%	
	Quite	4%	0%	11%	
	Very	0%	0%	0%	
Daily routine	Not	83%	72%	56%	0.243
	A little bit	10%	20%	22%	
	A bit	7%	4%	22%	
	Quite	0%	4%	0%	
	Very	0%	0%	0%	
Able to join in activities	Not	69%	52%	38%	0.135
	A little bit	24%	28%	38%	

For peer review only - http://bmjopen.bmj.com/site/about/guidelines.xhtml

A bit	3.5%	12%	13%
Quite	3.5%	8%	0%
Very	0%	0%	13%

[a]Comparing number of people experiencing "no" problems versus any problems (i.e., a little bit, a bit, quite and very) across GMFCS levels

For peer review only

For peer review only - http://bmjopen.bmj.com/site/about/guidelines.xhtml

STROBE 2007 (v4) Statement—Checklist of items that should be included in reports of *cross-sectional studies*

Section/Topic	Item #	Recommendation	Reported on page #
**Title and abstract**	1	(*a*) Indicate the study's design with a commonly used term in the title or the abstract	2
		(*b*) Provide in the abstract an informative and balanced summary of what was done and what was found	2
**Introduction**			
Background/rationale	2	Explain the scientific background and rationale for the investigation being reported	4
Objectives	3	State specific objectives, including any prespecified hypotheses	5
**Methods**			
Study design	4	Present key elements of study design early in the paper	5
Setting	5	Describe the setting, locations, and relevant dates, including periods of recruitment, exposure, follow-up, and data collection	5
Participants	6	(*a*) Give the eligibility criteria, and the sources and methods of selection of participants	5
Variables	7	Clearly define all outcomes, exposures, predictors, potential confounders, and effect modifiers. Give diagnostic criteria, if applicable	5-6
Data sources/ measurement	8*	For each variable of interest, give sources of data and details of methods of assessment (measurement). Describe comparability of assessment methods if there is more than one group	5-6
Bias	9	Describe any efforts to address potential sources of bias	
Study size	10	Explain how the study size was arrived at	7
Quantitative variables	11	Explain how quantitative variables were handled in the analyses. If applicable, describe which groupings were chosen and why	6
Statistical methods	12	(*a*) Describe all statistical methods, including those used to control for confounding	6
		(*b*) Describe any methods used to examine subgroups and interactions	
		(*c*) Explain how missing data were addressed	
		(*d*) If applicable, describe analytical methods taking account of sampling strategy	
		(*e*) Describe any sensitivity analyses	
**Results**			

For peer review only

Participants	13*	(a) Report numbers of individuals at each stage of study—eg numbers potentially eligible, examined for eligibility, confirmed eligible, included in the study, completing follow-up, and analysed	7
		(b) Give reasons for non-participation at each stage	7
		(c) Consider use of a flow diagram	
Descriptive data	14*	(a) Give characteristics of study participants (eg demographic, clinical, social) and information on exposures and potential confounders	7
		(b) Indicate number of participants with missing data for each variable of interest	7
Outcome data	15*	Report numbers of outcome events or summary measures	7
Main results	16	(*a*) Give unadjusted estimates and, if applicable, confounder-adjusted estimates and their precision (eg, 95% confidence interval). Make clear which confounders were adjusted for and why they were included	Tables
		(*b*) Report category boundaries when continuous variables were categorized	
		(*c*) If relevant, consider translating estimates of relative risk into absolute risk for a meaningful time period	
Other analyses	17	Report other analyses done—eg analyses of subgroups and interactions, and sensitivity analyses	
**Discussion**			
Key results	18	Summarise key results with reference to study objectives	8
Limitations	19	Discuss limitations of the study, taking into account sources of potential bias or imprecision. Discuss both direction and magnitude of any potential bias	11
Interpretation	20	Give a cautious overall interpretation of results considering objectives, limitations, multiplicity of analyses, results from similar studies, and other relevant evidence	8
Generalisability	21	Discuss the generalisability (external validity) of the study results	11
**Other information**			
Funding	22	Give the source of funding and the role of the funders for the present study and, if applicable, for the original study on which the present article is based	12

*Give information separately for cases and controls in case-control studies and, if applicable, for exposed and unexposed groups in cohort and cross-sectional studies.

Note: An Explanation and Elaboration article discusses each checklist item and gives methodological background and published examples of transparent reporting. The STROBE checklist is best used in conjunction with this article (freely available on the Web sites of PLoS Medicine at http://www.plosmedicine.org/, Annals of Internal Medicine at http://www.annals.org/, and Epidemiology at http://www.epidem.com/). Information on the STROBE Initiative is available at www.strobe-statement.org.

For peer review only

BMJ Open

A comparison of the CHU-9D and the EQ-5D-Y instruments in children and young people with cerebral palsy.

Journal:	*BMJ Open*
Manuscript ID	bmjopen-2020-037089.R1
Article Type:	Original research
Date Submitted by the Author:	14-May-2020
Complete List of Authors:	Ryan, Jennifer; Brunel University, College of Health and Life Sciences; Royal College of Surgeons in Ireland, Department of Public Health and Epidemiology<br>McKay, Ellen; Royal College of Surgeons in Ireland, Department of Public Health and Epidemiology<br>Anokye, Nana; Brunel University, Health Economics Research Group<br>Noorkoiv, Marika<br>Theis, Nicola; University of Gloucestershire, School of Sport and Exercise<br>Lavelle, Grace; King's College London, Institute of Psychiatry
**Primary Subject Heading**:	Health economics
Secondary Subject Heading:	Paediatrics, Neurology
Keywords:	HEALTH ECONOMICS, Developmental neurology & neurodisability < PAEDIATRICS, PAEDIATRICS

SCHOLARONE™
Manuscripts

I, the Submitting Author has the right to grant and does grant on behalf of all authors of the Work (as defined in the below author licence), an exclusive licence and/or a non-exclusive licence for contributions from authors who are: i) UK Crown employees; ii) where BMJ has agreed a CC-BY licence shall apply, and/or iii) in accordance with the terms applicable for US Federal Government officers or employees acting as part of their official duties; on a worldwide, perpetual, irrevocable, royalty-free basis to BMJ Publishing Group Ltd ("BMJ") its licensees and where the relevant Journal is co-owned by BMJ to the co-owners of the Journal, to publish the Work in this journal and any other BMJ products and to exploit all rights, as set out in our licence.

The Submitting Author accepts and understands that any supply made under these terms is made by BMJ to the Submitting Author unless you are acting as an employee on behalf of your employer or a postgraduate student of an affiliated institution which is paying any applicable article publishing charge ("APC") for Open Access articles. Where the Submitting Author wishes to make the Work available on an Open Access basis (and intends to pay the relevant APC), the terms of reuse of such Open Access shall be governed by a Creative Commons licence – details of these licences and which Creative Commons licence will apply to this Work are set out in our licence referred to above.

Other than as permitted in any relevant BMJ Author's Self Archiving Policies, I confirm this Work has not been accepted for publication elsewhere, is not being considered for publication elsewhere and does not duplicate material already published. I confirm all authors consent to publication of this Work and authorise the granting of this licence.

For peer review only

A comparison of the CHU-9D and the EQ-5D-Y instruments in children and young people with cerebral palsy.

Jennifer M. Ryan[1,2], Ellen McKay[2], Nana Anokye[1], Marika Noorkoiv[1], Nicola Theis[3], Grace Lavelle[4]

[1]College of Health and Life Sciences, Brunel University London, United Kingdom

[2]Department of Public Health and Epidemiology, RCSI, Ireland

[3]School of Sport and Exercise, University of Gloucestershire, Gloucester, United Kingdom

[4]Institute of Psychiatry, Psychology and Neuroscience, King's College London, United Kingdom

Corresponding author: Jennifer M. Ryan, College of Health and Life Sciences, Brunel University London, United Kingdom. Email: jennifer.ryan@brunel.ac.uk

Word count: 4207

For peer review only - http://bmjopen.bmj.com/site/about/guidelines.xhtml

Abstract

Objective: To compare the performance of the EuroQol 5D-youth (EQ-5D-Y) and child health utility 9D (CHU-9D) for assessing health-related quality of life (HRQoL) in children and young people (CYP) with cerebral palsy (CP).

Design: Cross-sectional study

Setting: England

Participants: Sixty-four CYP with cerebral palsy aged 10-19 years in Gross Motor Function Classification System (GMFCS) levels I-III.

Main outcome measures: Missing data were examined to assess feasibility. Associations between utility values and individual dimensions on each instrument were examined to assess convergent validity. Associations between utility values and GMFCS level were examined to assess known-group differences.

Results: Missing data were <5% for both instruments. Twenty participants (32.3%) and 11 participants (18.0%) reported full health for the EQ-5D-Y and CHU-9D, respectively. There was poor agreement between utilities from the two instruments (ICC=0.62; 95% limits of agreement -0.58 to 0.29). Correlations between EQ-5D-Y and CHU-9D dimensions were weak to moderate (r=0.25 to r=0.59). GMFCS level was associated with EQ-5D-Y utility values but not CHU-9D utility values.

Conclusions: The EQ-5D-Y and CHU-9D are feasible measures of HRQoL in CYP with CP. However, the two instruments demonstrate poor agreement and should not be used to measure and value HRQoL in CYP with CP interchangeably. We propose the CHU-9D may be preferable to use in this population as it assesses concepts that influence HRQoL among CYP with CP and provides less extreme utility values than the EQ-5D-Y.

Keywords: health economics; developmental neurology and neurodisability; paediatrics; cerebral palsy; EQ-5D-Y, CHU-9D

For peer review only

For peer review only - http://bmjopen.bmj.com/site/about/guidelines.xhtml

Strengths and limitations of this study

- This is the first study to compare the performance of the EQ-5D-Y and CHU-9D in a clinical population.
- Children and young people were recruited from the National Health Service, education and disability organisations across England.
- The findings are limited by a small sample and by inclusion of ambulatory individuals only.

For peer review only

For peer review only - http://bmjopen.bmj.com/site/about/guidelines.xhtml

Cerebral palsy (CP) is a heterogeneous disorder characterised by abnormal movement and posture. It is often co-existent with epilepsy, intellectual disability, and language, communication, or behavioural difficulties [1]. Its prevalence is 2 per 1,000 live births [2]; approximately 110,000 people live with CP in the UK [3]. Cerebral palsy is a lifelong condition. Management of CP encompasses medical, surgical and rehabilitation interventions [1]. While a large volume of research has examined the effectiveness of interventions for people with CP [4] there is limited research examining cost-effectiveness of such interventions.

Economic evaluation is used to inform the efficient allocation of resources in a healthcare setting. The cost utility analysis (CUA) is the type of economic evaluation recommended by the National Institute for Health and Care Excellence (NICE) particularly for interventions funded by NHS and personal social services [5]. The CUA commonly describes the relationship between costs and health benefits as the cost per quality-adjusted life year (QALY). QALYs incorporate both quantity and quality of life. QALYs are commonly calculated using an assessment of health-related quality of life (HRQoL) obtained from a preference-based instrument. Such instruments can provide a health state utility value, where 0 indicates a health state of equivalent value to being dead and 1.0 indicates full health, by applying a pre-specified algorithm based on preferences for health status identified in a specific population [6].

Generic HRQoL instruments are recommended for use in economic evaluations as they allow comparison across health care interventions and populations. However, if scores from generic measures differ, estimates of cost-effectiveness will be impacted, leading to uncertain conclusions regarding whether an intervention provides value for money. A number of generic measures have been used to obtain health state utility values from paediatric populations [7]. The most commonly used are the EuroQol 5D (EQ-5D), the Child Health Utility 9D (CHU-9D), the EuroQol 5D Youth version (EQ-5D-Y) and the Health Utilities Index 2 and 3 (HUI-2 and HUI-3) [7]. To date, however, only the HUI-2 and HUI-3 have been used to elicit utility values from children and young people (CYP) with CP and significant variation was reported in utility values [8].

The EQ-5D-Y and CHU-9D are two generic preference-based HRQoL instruments that were designed specifically for young people. The EQ-5D-Y is a youth modified version of the adult instrument, the EQ-5D, which was developed by revising the content and wording of the adult instrument [9]. The CHU-9D was developed from the outset for young people based on in-depth interviews with young people with chronic and acute health conditions [10]. Although the performance of the EQ-5D-Y and

For peer review only - http://bmjopen.bmj.com/site/about/guidelines.xhtml

CHU-9D has been examined in an adolescent population [11], their performance has not been examined among CYP with CP.

Given the financial cost of CP per annum is approximately AU$1.5 billion [12] and a wide range of interventions are currently available for people with CP [4], there is an increasing need for economic evaluation in this area. Prior to conducting economic evaluations, the performance of the EQ-5D-Y and CHU-9D in CYP with CP requires evaluation. The aim of this study was to compare the performance of the EQ-5D-Y and CHU-9D for assessing HRQoL in a community-based sample of CYP with CP. Specific objectives were to examine the feasibility of administering the instruments, to examine convergent validity, and to examine known-group differences for both measures.

Methods

Sample

CYP with CP who participated in a randomised controlled trial examining the effects of progressive resistance training were included in this study [13]. Data collected at baseline were used for this cross-sectional study. Participants were recruited from eight National Health Service (NHS) trusts in England, a special education needs school, a University and a primary care organisation in London, national organisations for people with disabilities, and by word of mouth. Inclusion criteria for participation in the trial were: aged 10-19 years with spastic CP and the ability to walk independently with or without a mobility aid (i.e., Gross Motor Function Classification System [GMFCS] levels I-III). Exclusion criteria for participation in the trial were: orthopaedic surgery of the lower limbs in the past 12 months, botulinum toxin type A injections or serial casting in the past 6 months, and insufficient cognition to comply with assessment procedures and the training programme. The study was approved by Brunel University London’s College of Health and Life Sciences Research Ethics Committee and the Surrey Borders Research Ethics Committee (ref: 15/LO/0843). Participants 16 years and older provided written consent. Those under 16 years provided assent alongside written consent from a parent or guardian.

Data on the person’s demographics, condition and HRQoL were collected using standardised questionnaires during an interview with a researcher at one time-point. Both HRQoL questionnaires were self-administered to all participants using the standardised instructions accompanying each measure. Assistance was provided by the researcher to read the questions if required. Further, the young person was allowed to ask their parent/guardian or researcher for assistance to answer the questions if required. Anatomical distribution was described as unilateral or bilateral [14]. Functional

For peer review only - http://bmjopen.bmj.com/site/about/guidelines.xhtml

mobility was classified according to the GMFCS. The GMFCS is a five-level classification system, where level I indicates most able and level V indicates most limited. Those in GMFCS level I are able to walk and run and climb stairs without assistance. Those in level II are able to walk in most settings but may use a hand-held mobility device indoors or wheeled mobility to travel long distances. Those in level III can walk using a hand-held mobility device but use a wheelchair or powered mobility outdoors. Participants selected a statement that best described their mobility based on descriptors of each GMFCS level [15]. Two physiotherapists retrospectively cross-referenced subjective ratings of GMFCS level against video recordings of participants, obtained as part of the baseline assessment.

Utility measurement

The EQ-5D-Y assesses a person's health across five dimensions. The five dimensions are "mobility", "looking after myself", "doing usual activities", "having pain or discomfort" and "feeling worried, sad or unhappy" [9]. Each dimension is rated on one of three levels (no problems, some problems, and a lot of problems) that describes a person's health today [9]. The EQ-5D-Y was developed by reviewing the applicability of the EQ-5D domain concepts and wording for children and adolescents [9]. The EQ-5D-Y is suitable for use in young people aged 8-19 years [16]. At present, there is no value set for the EQ-5D-Y. Although use of the adult value set (EQ-5D) for the EQ-5D-Y is not recommended, we calculated utilities using UK-based adult weights [17]. We acknowledge the limitation of this, but considered it to be the best method given the lack of weights for the EQ-5D-Y. Further, although the performance of the EQ-5D-Y using the adult value set has been examined in CYP with typical development [18], it has not been examined in a clinical population.

There are 9 dimension in the CHU-9D: "worried", "sad", "pain", "tired", "annoyed", "schoolwork", "sleep", "daily routine" and "ability to join in activities" [10]. For each dimension, the person describes how they are today according to one of five levels based on severity (e.g., not worried, a little bit worried, a bit worried, quite worried, very worried), which were determined from in-depth interviews with young people [10]. The CHU-9D is suitable for use in young people aged 7-17 years [10, 19]. The scoring algorithm based on the preferences of the UK adult general population was used to estimate utilities [20]. This method has been shown to be appropriate to use for young people [18].

Patient and Public Involvement

For peer review only

CYP with and without CP were involved in the design, conduct and dissemination plans of our research relating to the randomised controlled trial examining the effects of progressive resistance training.

Statistical analysis

The distribution of data was examined using histograms, Q-Q plots, and cross-tabulations. Mean and standard deviation (SD), median and interquartile range (IQR), frequencies and percentages were used to report the data as appropriate. We examined feasibility by reporting the number of participants with missing data and the percentage of missing data for each instrument. The instrument was considered feasible if missing data were <5% [21]. Participants who were missing data for an individual dimension of the EQ-5D-Y or CHU-9D were excluded from the calculation of utility values and from analyses involving that dimension.

To assess convergent validity, we calculated an intraclass correlation coefficient (ICC) between CHU-9D and EQ-5D-Y utility values. We interpreted an ICC >0.75 as indicating good agreement [22]. We compared mean utility between instruments using linear regression with a bootstrap procedure as there was evidence that residuals were not normally distributed. Bias corrected and accelerated bootstrap confidence intervals (CIs) were calculated from 2,000 replicates [23]. We also produced a Bland-Altman plot with 95% limits of agreement to examine agreement between utilities from the two instruments. We examined the association between 1) CHU-9D utility value and levels of each EQ-5D-Y dimension, and 2) EQ-5D-Y utility value and levels of each CHU-9D dimension, by calculating Spearman's correlation coefficients. We also examined associations between each dimension of the EQ-5D-Y and CHU-9D by calculating Spearman's correlation coefficients. Based on the description of each dimension and associations observed among adolescents with typical development [11], we hypothesised that the following dimensions would be correlated between the CHU-9D and EQ-5D-Y, respectively: "worried" vs. "feeling worried, sad or unhappy", "sad" vs. "feeling worried, sad or unhappy", "pain" vs "having pain/discomfort", "daily routine" vs. "doing usual activities", "daily routine" vs. "looking after myself", "able to join in" vs. "doing usual activities", and ""able to join in" vs. "mobility". To aid interpretation, we proposed a correlation of 0.10-0.39 to indicate a weak association, a correlation of 0.40-0.75 to indicate a moderate association, and a correlation of >0.75 to indicate a strong association [22,24]. However, this interpretation should be used with caution given that cut-offs for interpreting correlation coefficients are arbitrary and may be inconsistent with other studies [24].

For peer review only - http://bmjopen.bmj.com/site/about/guidelines.xhtml

For peer review only

We examined known-group differences by fitting linear regression models using a bootstrap procedure to compare CHU-9D utility values and EQ-5D-Y utility values, respectively, across functional mobility as defined by the GMFCS. A bootstrap procedure was used as there was evidence that residuals were not normally distributed. For each model, utility value was the dependent variable and bias corrected and accelerated bootstrap CIs were calculated from 2,000 replicates [23]. We also compared the number of people experiencing no problems versus any problems for each CHU-9D and EQ-5D-Y dimension across GMFCS level, using a Chi^2 test. It was expected that CYP with better functional mobility would have higher utilities [25,26]. MedCalc version 19.2.0 was used to produce the Bland-Altman plot. All other statistical analyses were performed using Stata version 13.

Results

Sixty-four participants were recruited to the study. One person did not complete the EQ-5D-Y or CHU-9D. Therefore, 63 participants were included in the analysis. Table 1 describes participant characteristics. The mean±SD age was 13.7±2.5 yr. The majority of participants (86%) were in GMFCS levels I or II indicating a mild lower limb impairment. The majority of participants were White British (59%) and attended a mainstream school (71%).

For the EQ-5D-Y, one participant did not provide a response to the “looking after myself” dimension. For the CHU-9D, two participants did not provide a response to the schoolwork dimension and one participant did not provide a response to the ability to “join in activities” dimension. Utility values for the EQ-5D-Y were therefore calculated for 62 out of 64 participants (96.9%) and utility values for the CHU-9D were calculated for 61 out of 64 participants (95.3%). Percentage missing data was 1.9% for the EQ-5D-Y and 2.0% for the CHU-9D.

The distribution of EQ-5D-Y and CHU-9D utilities is shown in Figures 1 and 2. For the EQ-5D-Y and CHU-9D, respectively, 20 participants (32.3%) and 11 participants (18.0%) reported full health. The median (IQR) EQ-5D-Y utility value was 0.80 (0.62 to 1.00). The mean±SD EQ-5D-Y utility value was 0.73±0.29 (range -0.17 to 1.00). Two participants (3.2%) reported a “worse than death” health state for the EQ-5D-Y. The median (IQR) CHU-9D utility value was 0.92 (0.83 to 0.96). The mean±SD CHU-9D utility value was 0.89±0.10 (range 0.56 to 1.00). There was poor agreement between utilities from the two instruments as indicated by an ICC of 0.62. The CHU-9D utility value was on average 0.15 (95% CI 0.09 to 0.25) higher than the EQ-5D-Y utility value. 95% limits of agreement were -0.58 to 0.29 (Figure 3).

For peer review only - http://bmjopen.bmj.com/site/about/guidelines.xhtml

The percentages of reported problems across dimensions of the EQ-5D-Y and CHU-9D are presented in Tables 2 and 3. There was evidence of correlation between EQ-5D-Y utility values and level of severity for all dimensions of the CHU-9D except for the “sad” dimension. Correlations ranged from -0.27 to -0.64; Table 2). For all dimensions of the EQ-5D-Y, the median CHU-9D utility value decreased with increasing levels of severity on the EQ-5D-Y dimensions (Table 3). For all dimensions of the EQ-5D-Y, the majority of respondents reported themselves in the least severe level (i.e. no problems). However, responses for most dimensions of the EQ-5D-Y were spread across all levels, from no problems to a lot of problems. The exception to this was the “worried, sad or unhappy” dimension; no participant reported feeling very worried, sad or unhappy. Similarly, for all dimensions of the CHU-9D except for “tired”, the majority of participants reported themselves in the least severe level. However, responses were not spread across all levels for each dimension. For the “worried”, “sad”, “pain”, “annoyed”, “ “sleep” and “daily routine” dimensions of the CHU-9D, no participant reported the most severe level.

Correlations between CHU-9D and EQ-5D-Y dimensions are presented in Table 4. Moderate correlations (r=0.43-0.59) were observed for all hypothesised associations, except for “worried” vs. “feeling worried, sad or unhappy” (r=0.25) and “daily routine” vs. “doing usual activities” (r=0.35). Several unexpected correlations were observed between dimensions. Namely, “schoolwork” on the CHU-9D was associated with “mobility” (r=0.56), “pain or discomfort” (r=0.55), and “doing usual activities” (r=0.44) on the EQ-5D-Y; “tired” on the CHU-9D was associated with “pain/discomfort” on the EQ-5D-Y (r = 0.40) and “annoyed” on the CHU-9D was associated with. “worried/sad/unhappy” on the EQ-5D-Y (r = 0.53).

Median (IQR) utilities by GMFCS level are presented in Table 1. EQ-5D-Y utility value was associated with GMFCS level (R^2 = 0.231, p=0.016; supplemental Table 1). As expected, EQ-5D-Y utility value was on average 0.43 lower in individuals in level III compared to those in level I (95% CI 0.14 to 0.73) and 0.36 lower in individuals in level III compared to those in level II (95% CI 0.06 to 0.66). However, there was no difference in utility value between those in levels I and II. Although median CHU-9D utility value was lower in GMFCS level III compared to GMFCS level I and II (Table 1), there was no evidence that CHU-9D utility value was associated with GMFCS level (R^2 = 0.071, p=0.170; supplemental table 2). As presented in Table 5, the percentage of people reporting some or a lot of problems on the “mobility” and “looking after myself” dimensions of the EQ-5D-Y differed according to GMFCS level, with the percentage of people reporting problems increasing from GMFCS levels I to III. The percentage of people reporting problems was not associated with GMFCS level for any other

For peer review only - http://bmjopen.bmj.com/site/about/guidelines.xhtml

EQ-5D-Y dimension. There was also no evidence of an association between the percentage of people reporting problems and GMFCS level for any dimension of the CHU-9D (Table 6).

Discussion

The aim of this study was to compare the performance of the EQ-5D-Y and CHU-9D for assessing HRQoL in CYP with CP. Specific objectives were to examine feasibility, convergent validity, and known-group differences. To our knowledge, this is the first study to compare the EQ-5D-Y and CHU-9D in a clinical population. Although the results indicate that the two instruments are feasible to use when administered by interview to CYP with CP, the two instruments have poor agreement and may not be used interchangeably to measure and value HRQoL among CYP with CP. The EQ-5D-Y utility value was on average 0.15 lower than the CHU-9D utility value. However, there was considerable variation in individual differences between instruments. 95% limits of agreement demonstrated that the EQ-5D-Y utility value may be 0.29 higher or 0.58 lower than the CHU-9D utility value for an individual. Additionally, dimensions on each instrument that were hypothesised to measure similar concepts were only weakly to moderately associated.

Two studies compared the EQ-5D-Y and CHU-9D in CYP with typical development [11,18]. Agreement between EQ-5D-Y and CHU-9D utility values was better among adolescents with typical development than among CYP with CP, with an ICC of 0.80 and much narrower 95% limits of agreement (-0.268 to 0.241) reported [11]. Although the median EQ-5D-Y utility value among CYP in this study was similar to that reported for Australian adolescents with typical development (0.80 versus 0.83), the median CHU-9D utility was higher (0.92 versus 0.83) [11]. The median CHU-9D utility reported by CYP with CP was more similar to that reported by children, aged 6-7 years, with typical development living in England (0.92 versus 0.90) and the median EQ-5D-Y utility was identical between these groups [18]. This may be because the study of children living in England used the same algorithms to estimate utilities as the current study, while the study of adolescents used algorithms developed in the Australian population. It is also plausible that CYP with CP have better HRQoL than CYP with typical development. A study of a large sample of CYP with CP across Europe found that CYP with CP had better QoL in five domains (moods and emotions, self-perception, autonomy, relationships with parents, school life) compared to CYP in the general population matched for age, sex and country [27].

Although the EQ-5D-Y utility value was on average lower than the CHU-9D utility value, 32% of participants reported full health when using the EQ-5D-Y compared to only 18% of participants when

For peer review only - http://bmjopen.bmj.com/site/about/guidelines.xhtml

using the CHU-9D. A similar ceiling effect for the EQ-5D-Y was reported among CYP with typical development [11,18]. Only 18% of CYP with CP reported full health when using the CHU-9D. Although a large proportion of CYP had "perfect health" according to the EQ-5D-Y, two children reported a health state worse than death. Neither of these children reported the worst possible health state according to the CHU-9D. The CHU-9D may be more sensitive to varying severities of health because of the larger number of levels for each dimension. The CHU-9D is also the only generic measure that was specifically designed for CYP and the levels were determined from in-depth interviews with young people [10]. Further, as suggested previously, the extreme health states observed for the EQ-5D-Y may be a result of misapplication of the adult EQ-5D tariff to the health states defined by the EQ-5D-Y [18]. The findings from this study provide additional evidence that the EQ-5D-Y should not be used to measure utilities until a specific value set for the EQ-5D-Y is available.

Although we found associations between a number of dimensions on the EQ-5D-Y and CHU-9D as hypothesised, these associations were only weak to moderate, despite both instruments being administered at the same time-point, by the same researcher and in the same environment. These correlations were weaker than those reported among Australian adolescents with typical development [11]. In particular, the correlation between "feeling worried, sad or unhappy" on the EQ-5D-Y and "worried" on the CHU-9D was 0.70 among adolescents with typical development, compared to 0.25 among CYP with CP. This suggests that CYP with CP interpret these two dimensions differently and they do not measure the same concept in this population. The strongest correlation between EQ-5D-Y and CHU-9D dimensions among adolescents with typical development was between "having pain or discomfort" and "pain" ($r=0.753$) [11]. However, "having pain or discomfort" and "pain" were only moderately correlated in CYP with CP ($r=0.47$). Many CYP with CP experience frequent pain [24], and as a result, their interpretation of the EQ-5D-Y question about pain and discomfort may differ to their interpretation of the CHU-9D question about pain only. Indeed, fifty-six percent of CYP with CP reported no pain or discomfort on the EQ-5D-Y, while 62% reported no pain on the CHU-9D. Of the hypothesised correlations, only the correlation between "looking after myself" on the EQ-5D-Y and "daily routine" on the CHU-9D was stronger among CYP with CP compared to adolescents with typical development [11]. As the "looking after myself" dimension refers specifically to washing and dressing, this suggests that CYP with CP interpret self-care as part of their daily routine.

For peer review only - http://bmjopen.bmj.com/site/about/guidelines.xhtml

We also observed a number of unexpected associations between EQ-5D-Y and CHU-9D dimensions. “Schoolwork” on the CHU-9D was associated with “mobility”, “having pain or discomfort” and “doing usual activities” on the EQ-5D-Y. Approximately 45% of CYP with CP have an intellectual disability, which may range from mild to severe [28]. Although we excluded individuals with insufficient cognition to comply with assessment procedures, some participants may have had a mild intellectual disability. As CYP with intellectual disability are likely to have more severely impaired physical functioning [28], it is possible that CYP with intellectual disability have more problems completing schoolwork and more problems with mobility and doing usual activities. Alternatively, CYP with more severe physical impairment, who will have more problems with mobility and doing usual activities, may also have more problems with completing schoolwork as a result of their physical impairment.

We observed an association between EQ-5D-Y utility values and GMFCS level but not between CHU-9D utility values and GMFCS level. Two studies reported that utility values obtained from the HUI-3 differed according to GMFCS level [25,26]. However, regardless of the instrument used, we found that those in GMFCS levels I and II have a similar mean utility value, while those in GMFCS level III have a lower mean utility value compared to levels I and II. The lack of statistical evidence of an association between CHU-9D utility value and GMFCS level may be due to the narrower utility range of the CHU-9D, which resulted in smaller incremental changes between levels. There was also a small number of participants in GMFCS level III, which likely resulted in reduced statistical power to detect differences between groups. However, it is also plausible that HRQoL is not associated with functional mobility. When condition-specific measures of QoL were used, associations between QoL and GMFCS level were not observed [26,27].

The mean EQ-5D-Y utility value for each GMFCS level was similar to values obtained from the HUI-3 in one study (0.84, 0.50 and 0.39 for adolescents in GMFCS levels I, II and III, respectively) [26] but not similar to those in a second study (0.67, 0.59 and 0.43 in GMFCS levels I, II and III, respectively) [25]. The mean CHU-9D utility values for each GMFCS level were higher than those obtained from the HUI-3 [25,26]. Discrepancies may be due to differences in the algorithms used to derive utility values. HUI-3 utility values were derived in one study using preferences from the general adult population in Canada [26]. The second study did not state the preferences used to derive HUI-3 utility values [25]. Differences in utilities between the CHU-9D and HUI-3 do not necessarily indicate that the CHU-9D is inaccurate. The HUI-3 was only weakly correlated with a condition-specific measure of HRQoL in CYP with CP [26]. This weak correlation highlights that condition-specific

For peer review only - http://bmjopen.bmj.com/site/about/guidelines.xhtml

measures and generic measures of HRQoL may not assess similar concepts. However, unlike generic measures, condition-specific measures are not recommended for evaluations across different conditions [29]. Although a generic measure, the CHU-9D may be more likely than the EQ-5D-Y and HUI to capture dimensions of HRQoL that are important to CYP with CP. In particular, the CHU-9D includes more questions about psychological difficulties and pain, which are associated with QoL among CYP with CP [27].

Limitations

The findings of this study are limited by a small sample. In particular there were a small number of participants in GMFCS level III. Although the sample included CYP with CP in GMFCS levels I-III, which represents about 70% of individuals with CP [30], the findings may not be applicable to non-ambulatory CYP. As the sample volunteered to participate, they may have a higher HRQoL than those who did not volunteer.

In conclusion, the findings of this study illustrate that the EQ-5D-Y and the CHU-9D are feasible to use among CYP with CP. However, there is poor agreement between utility values elicited from the two instruments and they should not be used interchangeably to measure and value HRQoL in CYP with CP. This study provides further evidence that it is not appropriate to use the adult EQ-5D tariff to derive utility values from the EQ-5D-Y. Additionally, we propose that the CHU-9D is preferable to the EQ-5D-Y for measuring HRQoL among CYP with CP because it was developed based on interviews with CYP, it assesses concepts that influence QoL among CYP with CP, and produces less extreme values than the EQ-5D-Y. This study also demonstrates that agreement between the EQ-5D-Y and CHU-9D is poorer among CYP with CP compared to CYP with typical development, suggesting that these instruments require evaluation in other clinical populations.

Competing interests: None declared.

Author statement: JR and GL conceived the study. JR, GL and NA designed the study. GL, JR, MN and NT acquired data. JR, GL and EM performed the analysis. NA contributed to analysis and interpretation of the data. JR, GL and EM drafted the manuscript. NA, MN and NT critically revised the manuscript. All authors approved the final manuscript.

Data sharing statement: Data are available upon reasonable request.

Ethics approval: The study was approved by Brunel University London's College of Health and Life Sciences Research Ethics Committee and the Surrey Borders Research Ethics Committee (ref: 15/LO/0843).

For peer review only - http://bmjopen.bmj.com/site/about/guidelines.xhtml

Funding: Action Medical Research and the Chartered Society of Physiotherapy Charitable Trust have jointly funded this project, and it is supported by a generous grant from The Henry Smith Charity (GN2340).

References

1. Colver A, Fairhurst C, Pharoah PO. Cerebral palsy. *Lancet* 2014;383:1240-9.
2. Oskoui M, Coutinho F, Dykeman J, *et al.* An update on the prevalance of cerebral palsy: a systematic review and meta-analysis. *Dev Med Child Neurol* 2013;55:509-19.
3. Department of Health Long-term Conditions National Service Framework Team. The National Service Framework for Long-Term Conditions. *Department of Health* 2005.
4. Novak I, McIntyre S, Morgan C, *et al.* A systematic review of interventions for children with cerebral palsy: state of the evidence. *Dev Med Child Neurol* 2013;55:885-910.
5. NICE. Developing NICE guidelines: the manual. London; 2018.
6. Whitehead SJ, Ali S. Health outcomes in economic evaluation: the QALY and utilities. *Br Med Bull* 2010;96:5-21.
7. Thorrington D, Eames K. Measuring Health Utilities in Children and Adolescents: A Systematic Review of the Literature. *PloS One,* 2015;10:e0135672.
8. Tonmukayakul U, Le LK, Mudiyanselage SB, *et al.* A systematic review of utility values in children with cerebral palsy. *Qual Life Res* 2019;28:1-12.
9. Wille N, Badia X, Bonsel G, *et al.* Development of the EQ-5D-Y: a child-friendly version of the EQ-5D. *Qual Life Res* 2010;19:875-86.
10. Stevens K. Developing a descriptive system for a new preference-based measure of health-related quality of life for children. *Qual Life Res* 2009;18:1105-13.
11. Chen G, Flynn T, Stevens K, *et al.* Assessing the Health-Related Quality of Life of Australian Adolescents: An Empirical Comparison of the Child Health Utility 9D and EQ-5D-Y Instruments. *Value Health* 2015;18:432-8.
12. Access Economics. The economic impact of cerebral palsy in Australia in 2007. Canberra, Australia: Access Economics 2008.
13. Ryan JM, Theis N, Kilbride C, *et al.* Strength Training for Adolescents with cerebral palsy (STAR): study protocol of a randomised controlled trial to determine the feasibility, acceptability and efficacy of resistance training for adolescents with cerebral palsy. *BMJ open.* 2016;6:e012839.
14. Rosenbaum P, Paneth N, Leviton A, *et al.* A report: the definition and classification of cerebral palsy April 2006. *Dev Med Child Neurol Suppl* 2007;109:8-14.
15. Palisano R, Rosenbaum P, Walter S, *et al.* Development and reliability of a system to classify gross motor function in children with cerebral palsy. *Dev Med Child Neurol* 1997;39:214-23.
16. Ravens-Sieberer U, Wille N, Badia X, *et al.* Feasibility, reliability, and validity of the EQ-5D-Y: results from a multinational study. *Qual Life Res* 2010;19:887-97.
17. Dolan P. Modelling valuations for EuroQol health states. *Med Care* 1997;35:1095-108.
18. Canaway AG, Frew EJ Measuring preference-based quality of life in children aged 6-7 years: a comparison of the performance of the CHU-9D and EQ-5D-Y--the WAVES pilot study. *Qual Life Res* 2013;22:173-83.
19. Ratcliffe J, Stevens K, Flynn T, *et al.* An assessment of the construct validity of the CHU9D in the Australian adolescent general population. *Qual Life Res* 2012;21:717-25.
20. Stevens K. Valuation of the Child Health Utility 9D Index. *Pharmacoeconomics* 2012;30:729-747.
21. Todd D. Little, Terrence D. Jorgensen, Kyle M. Lang,1, and E. Whitney G. Moore. On the Joys of Missing Data. *Journal of Pediatric Psychology* 39(2) pp. 151–162, 2014 doi:10.1093/jpepsy/jst048
22. Fleiss JL, CohenJ. The equivalence of weighted kappa and the intraclass correlation coefficient as measures of reliability. *Educ Psychol Meas* 1973;33:613–9.

For peer review only - http://bmjopen.bmj.com/site/about/guidelines.xhtml

23. Good P. Permutation, Parametric, and Bootstrap Tests of Hypotheses. Third edition. 2005 Springer.
24. Schober P, Boer C, Schwarte LA. Correlation Coefficients: Appropriate Use and Interpretation. *Anesth Analg*. 2018;126:1763-8.
25. Young NL, Rochon TG, McCormick A, *et al.* The health and quality of life outcomes among youth and young adults with cerebral palsy. *Arch Phys Med Rehabil* 2010;91:143-8.
26. Rosenbaum PL, Livingston MH, Palisano RJ, *et al.* Quality of life and health-related quality of life of adolescents with cerebral palsy. *Dev Med Child Neurol* 2007;49:516-21.
27. Colver A, Rapp M, Eisemann N, *et al.* Self-reported quality of life of adolescents with cerebral palsy: a cross-sectional and longitudinal analysis. *Lancet* 2015;385:705-16.
28. Reid SM, Meehan EM, Arnup SJ, *et al.* Intellectual disability in cerebral palsy: a population-based retrospective study. *Dev Med Child Neurol* 2018;60:687-94.
29. Seow LSE, Tan THG, Abdin E, *et al.* Comparing disease-specific and generic quality of life measures in patients with schizophrenia. *Psychiatry Res* 2019;273:387-93.
30. Children and young people with cerebral palsy in Northern Ireland (1981-2008). A comprehensive report from the Northern Ireland Cerebral Palsy Register. HSC Public Health Agency and Queen's University Belfast.

For peer review only

For peer review only - http://bmjopen.bmj.com/site/about/guidelines.xhtml

Table 1. Participant characteristics

	n (%)	Mean (SD)	Range	Mean (SD) EQ-5D-Y utility value	Median (IQR) EQ-5D-Y utility value	Mean (SD) CHU-9D utility value	Median (IQR) CHU-9D utility value
Age, yr	63	13.7 (2.5)	10-19	-	-	-	-
Female	26 (41.3)			0.77 (0.29)	0.83 (0.69-1.00)	0.90 (0.08)	0.92 (0.83-0.97)
Male	37 (58.7)			0.71 (0.29)	0.77 (0.62-0.93)	0.88 (0.11)	0.92 (0.81-0.95)
Height, cm	63	154.3 (12.7)	131.5-180.9	-	-	-	-
Mass, kg	63	49.3 (13.7)	27.4-78.5	-	-	-	-
**Ethnicity**							
White British	37 (58.7)			0.80 (0.20)	0.81 (0.69-1.00)	0.89 (0.09)	0.92 (0.84-0.95)
Black or Black British	5 (7.9)			0.62 (0.44)	0.81 (0.19-1.00)	0.90 (0.10)	0.90 (0.81-1.00)
Asian or Asian British	9 (14.3)			0.59 (0.49)	0.73 (0.20-1.00)	0.88 (0.12)	0.92 (0.89-0.95)
Other	12 (19.0)			0.69 (0.22)	0.75 (0.57-0.81)	0.87 (0.13)	0.87 (0.82-0.97)
**GMFCS level**							
I	29 (46.0)			0.82 (0.20)	0.81 (0.73-1.00)	0.89 (0.11)	0.92 (0.82-0.95)
II	25 (39.7)			0.75 (0.25)	0.75 (0.62-1.00)	0.90 (0.08)	0.92 (0.86-0.97)
III	9 (14.3)			0.39 (0.43)	0.44 (0.00-0.76)	0.82 (0.12)	0.83 (0.72-0.89)
**Distribution**							
Unilateral	31 (49.2)			0.81 (0.18)	0.80 (0.69-1.00)	0.91 (0.09)	0.92 (0.87-0.98)
Bilateral	32 (50.8)			0.66 (0.35)	0.75 (0.59-1.00)	0.87 (0.11)	0.87 (0.81-0.95)
**Type of School**							

For peer review only

For peer review only - http://bmjopen.bmj.com/site/about/guidelines.xhtml

Mainstream school	45 (71.4)	0.77 (0.24)	0.80 (0.67-1.00)	0.88 (0.08)	0.90 (0.82-0.95)
SEN	13 (20.6)	0.62 (0.41)	0.80 (0.44-0.85)	0.89 (0.11)	0.92 (0.86-0.98)
Further college education	2 (3.2)	0.85 (0.22)	0.85 (0.69-1.00)	0.99 (0.01)	0.99 (0.98-1.00)
University	3 (4.8)	0.67 (0.45)	0.85 (0.16-1.00)	0.85 (0.25)	1.00 (0.56-1.00)

SD: standard deviation; GMFM: Gross motor function classification system; SEN: Special Needs Education

For peer review only

Table 2. Number and percentage of participants reporting problems across EQ-5D-Y dimensions

EQ-5D-Y dimensions	levels	Frequency (%)	n	Median±IQR CHU-9D utility value	rho	p value
Mobility	No	50.8	32	0.95±0.09	-0.52	<0.001
	Some	44.4	28	0.84±0.13		
	A lot of	4.8	3	0.75±0.05		
Looking after myself	No	66.7	42	0.94±0.09	-0.46	<0.001
	Some	27.0	17	0.83±0.10		
	A lot of	4.8	3	0.72±0.27		
	Missing	1.6	1	0.78		
Usual activities	No	66.7	42	0.93±0.11	-0.54	<0.001
	Some	28.6	18	0.83±0.15		
	A lot of	4.8	3	0.72±0.16		
Pain or discomfort	No	55.6	35	0.95±0.11	-0.54	<0.001
	Some	38.1	24	0.86±0.16		
	A lot of	6.3	4	0.72±0.24		
Worried, sad or unhappy	Not	84.1	53	0.92±0.11	-0.44	<0.001
	A bit	15.9	10	0.79±0.05		
	Very	0	0	-		

For peer review only

For peer review only - http://bmjopen.bmj.com/site/about/guidelines.xhtml

Table 3. Number and percentage of participants reporting problems across CHU-9D dimensions

CHU-9D dimensions	levels	Frequency (%)	n	Median±IQR EQ-5D-Y utility value	rho	p value
Worried	Not worried	82.5	52	0.81±0.29	-0.38	0.002
	A little bit	15.9	10	0.55±0.66		
	A bit	1.6	1	-0.11		
	Quite	0	0	-		
	Very	0	0	-		
Sad	Not sad	93.7	59	0.80±0.34	-0.12	0.350
	A little bit	3.2	2	0.50±0.69		
	A bit	3.2	2	0.68±0.33		
	Quite	0	0	-		
	Very	0	0	-		
Pain	No pain	61.9	39	0.85±0.29	-0.42	<0.001
	A little bit	30.2	19	0.73±0.23		
	A bit	3.2	2	0.39±0.60		
	Quite	4.8	3	0.16±0.73		
	A lot	0	0	-		
Tired	Not tired	36.5	23	0.82±0.27	-0.44	<0.001
	A little bit	42.9	27	0.81±0.31		
	A bit	9.5	6	0.59±0.07		
	Quite	7.9	5	0.19±0.46		
	Very	3.2	2	0.45±0.52		
Annoyed	Not annoyed	84.1	53	0.81±0.30	-0.27	0.034
	A little bit	11.1	7	0.62±0.33		
	A bit	3.2	2	0.50±0.69		
	Quite	1.6	1	0.52		
	Very	0	0	-		

For peer review only - http://bmjopen.bmj.com/site/about/guidelines.xhtml

Schoolwork/homework	No problems	63.5	40	0.93±0.21	-0.64	<0.001
	Few problems	17.5	11	0.68±0.10		
	Some problems	14.3	9	0.55±0.52		
	Many problems	0	0	-		
	Can't do schoolwork	1.6	1	0.20		
	Missing	3.2	2	-		
Sleep	No problems	71.4	45	0.81±0.29	-0.38	0.002
	Few problems	20.6	13	0.70±0.24		
	Some problems	4.8	3	0.73±0.07		
	Many problems	3.2	2	0.02±0.27		
	Can't sleep	0	0	-		
Daily routine	No problems	74.6	47	0.81±0.27	-0.48	<0.001
	Few problems	15.9	10	0.52±0.59		
	Some problems	7.9	5	0.66±0.14		
	Many problems	1.6	1	0.09		
	Can't do daily routine	0	0	-		
Able to join in activities	Any activities	57.1	36	0.85±0.27	-0.43	<0.001
	Most activities	27.0	17	0.73±0.23		
	Some activities	7.9	5	0.60±0.33		
	Few activities	4.8	3	0.71±0.33		
	No activities	1.6	1	-0.11		
	Missing	1.6	1	-		

For peer review only

Table 4. Spearman rank correlation coefficients between CHU-9D and EQ-5D-Y dimensions.

EQ-5D-Y dimensions		CHU-9D dimensions								
		**Worried**	**Sad**	**Pain**	**Tired**	**Annoyed**	**Schoolwork**	**Sleep**	**Daily routine**	**Activities**
**Mobility**	*r*	***0.30***	*-0.01*	***0.35***	***0.36***	*0.19*	***0.56***[a]	***0.31***	*0.19*	***0.49***[b]
	p	**0.017**	0.950	**0.006**	**0.004**	0.140	**<0.001**	**0.013**	0.144	**<0.001**
**Looking after myself**	*r*	***0.26***[b]	*-0.04*[b]	*0.21*[b]	*0.22*[b]	*0.08*[b]	***0.39***[c]	***0.27***[b]	***0.59***[b]	***0.36***[a]
	p	**0.040**	0.741	0.098	0.086	0.525	**0.002**	**0.031**	**<0.001**	**0.004**
**Activities**	*r*	***0.36***	*0.08*	***0.30***	*0.23*	***0.25***	***0.44***[a]	***0.38***	***0.35***	***0.54***[b]
	p	**0.004**	0.543	**0.017**	0.065	**0.049**	**<0.001**	**0.002**	**0.006**	**<0.001**
**Pain or discomfort**	*r*	***0.33***	*0.07*	***0.47***	***0.40***	*0.22*	***0.55***[a]	***0.39***	***0.25***	***0.32***[b]
	p	**0.008**	0.600	**<0.001**	**0.001**	0.090	**<0.001**	**0.002**	**0.048**	**0.011**
**Worried, sad, unhappy**	*r*	***0.25***	***0.43***	*0.19*	***0.26***	***0.53***	***0.39***[a]	*-0.01*	***0.33***	*0.15*[b]
	p	**0.048**	**<0.001**	0.143	**0.039**	**<0.001**	**0.002**	0.944	**0.008**	0.239

Note: correlations with p value <0.05 highlighted in bold; n=63 for all analyses unless stated otherwise.
[a]n=61; [b]n=62; [c]n=60

For peer review only

Table 5. Percentage reporting problems on each EQ-5D-Y dimension across GMFCS level

EQ-5D-Y dimensions	levels	GMFCS level I	GMFCS level II	GMFCS level III	*p value*[a]
		n=29	n=25	n=9	
Mobility	No	69%	44%	11%	0.007
	Some	31%	56%	56%	
	A lot of	0%	0%	33%	
Looking after myself	No	83%	56%	50%	0.036
	Some	17%	40%	25%	
	A lot of	0%	4%	25%	
Usual activities	No	72%	72%	33%	0.072
	Some	28%	24%	44%	
	A lot of	0%	4%	22%	
Pain or discomfort	No	59%	60%	33%	0.348
	Some	38%	36%	44%	
	A lot of	3%	4%	22%	
Worried, sad or unhappy	No	79%	92%	78%	0.380
	Some	21%	8%	22%	
	A lot of	0%	0%	0%	

[a]Comparing number of people experiencing “no” problems versus any problems (i.e., some and a lot of) across GMFCS levels

For peer review only

Table 6. Percentage reporting problems on each CHU-9D dimension across GMFCS level

CHU-9D dimensions	levels	GMFCS level I	GMFCS level II	GMFCS level III	*p value*[a]
		n=29	n=25	n=9	
Worried	Not worried	83%	84%	78%	0.914
	A little bit	17%	16%	11%	
	A bit	0%	0%	11%	
	Quite	0%	0%	0%	
	Very	0%	0%	0%	
Sad	Not sad	86%	100%	100%	0.082
	A little bit	7%	0%	0%	
	A bit	7%	0%	0%	
	Quite	0%	0%	0%	
	Very	0%	0%	0%	
Pain	No pain	55%	72%	56%	0.408
	A little bit	38%	24%	22%	
	A bit	0%	4%	11%	
	Quite	7%	0%	11%	
	A lot	0%	0%	0%	
Tired	Not tired	31%	44%	33%	0.601
	A little bit	52%	32%	44%	
	A bit	7%	12%	11%	
	Quite	10%	8%	0%	

For peer review only

	Very	0%	4%	11%	
Annoyed	Not annoyed	72%	96%	89%	0.056
	A little bit	17%	4%	11%	
	A bit	7%	0%	0%	
	Quite	4%	0%	0%	
	Very	0%	0%	0%	
Schoolwork/homework	No problems	72%	64%	43%	0.104
	Few problems	17%	16%	29%	
	Some problems	10%	20%	14%	
	Many problems	0%	0%	14%	
	Can't do schoolwork	0%	0%	0%	
Sleep	Not	79%	68%	56%	0.343
	A little bit	10%	28%	33%	
	A bit	7%	4%	0%	
	Quite	4%	0%	11%	
	Very	0%	0%	0%	
Daily routine	Not	83%	72%	56%	0.243
	A little bit	10%	20%	22%	
	A bit	7%	4%	22%	
	Quite	0%	4%	0%	
	Very	0%	0%	0%	
Able to join in activities	Not	69%	52%	38%	0.135
	A little bit	24%	28%	38%	

For peer review only - http://bmjopen.bmj.com/site/about/guidelines.xhtml

For peer review only

A bit	3.5%	12%	13%
Quite	3.5%	8%	0%
Very	0%	0%	13%

[a]Comparing number of people experiencing “no” problems versus any problems (i.e., a little bit, a bit, quite and very) across GMFCS levels

For peer review only

For peer review only - http://bmjopen.bmj.com/site/about/guidelines.xhtml

Figure legend

Figure 1. Distribution of utility values for the EQ-5D-Y.

Figure 2. Distribution of utility values for the CHU-9D.

Figure 3. Bland-Altman plot of differences between EQ-5D-Y and CHU-9D utility values against average of EQ-5D-Y and CHU-9D utility values.

For peer review only

For peer review only - http://bmjopen.bmj.com/site/about/guidelines.xhtml

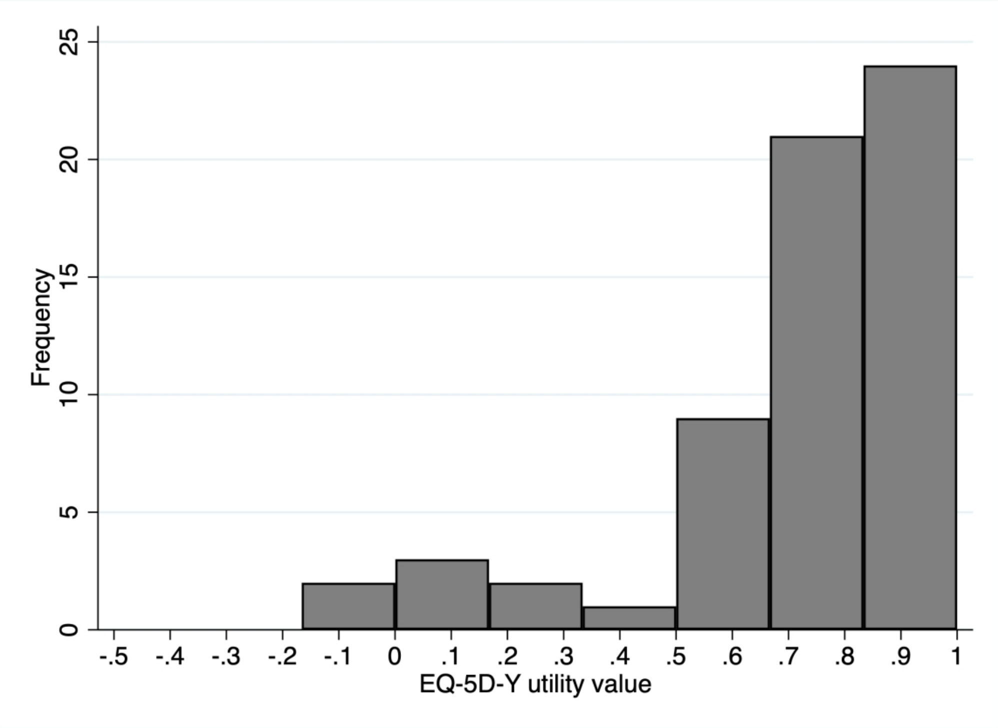

Figure 1. Distribution of utility values for the EQ-5D-Y.

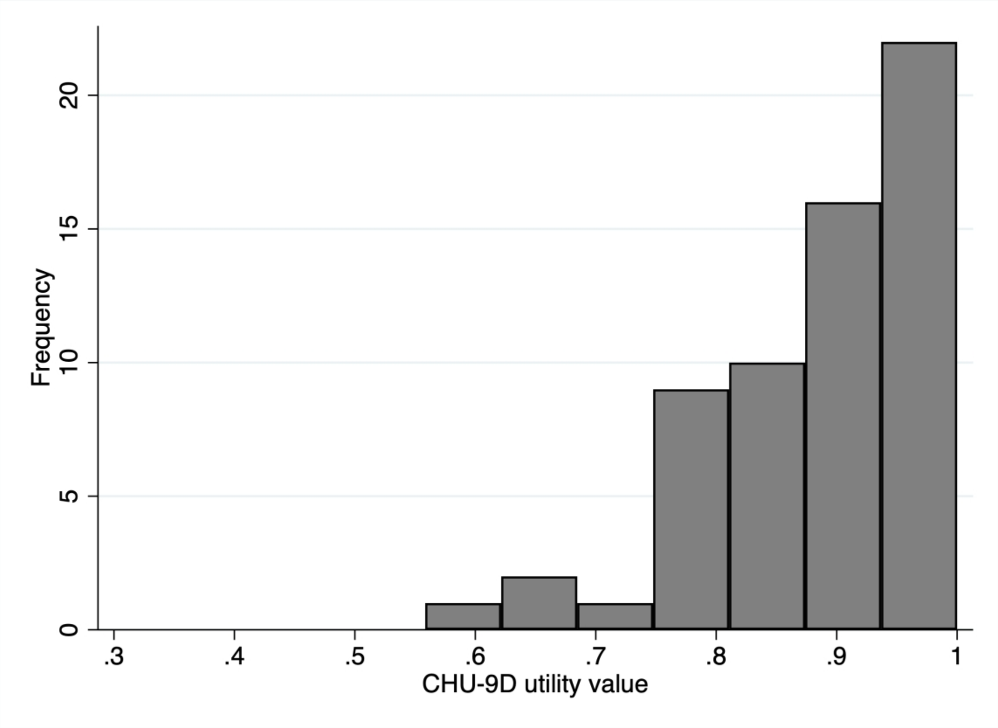

Figure 2. Distribution of utility values for the CHU-9D.

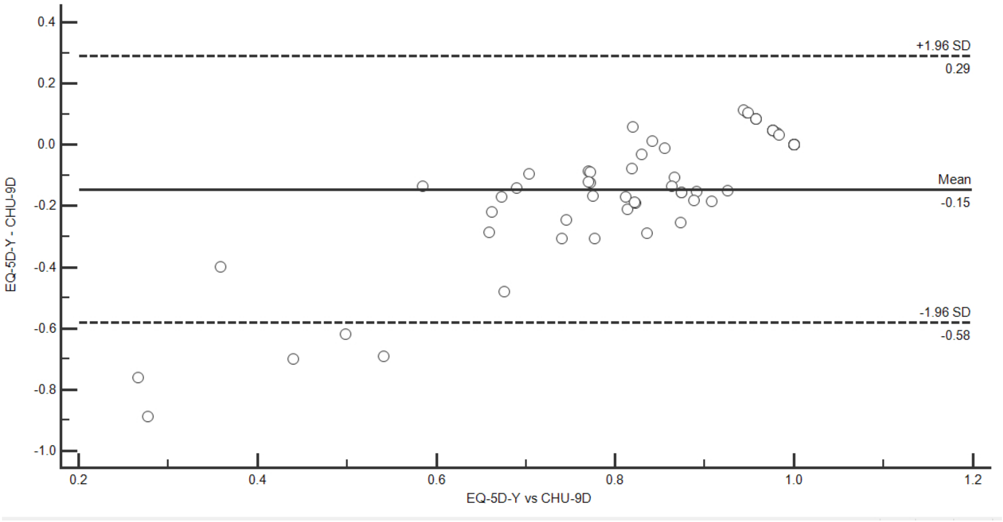

Figure 3. Bland-Altman plot of differences between EQ-5D-Y and CHU-9D utility values against average of EQ-5D-Y and CHU-9D utility values.

Supplemental Table 1. Linear regression analysis examining association between EQ-5D-Y utility values (dependent variable) and GMFCS level.

	Coefficient	Bootstrap SE	95% CI[a]	R^2	p value
GMFCS level I	reference	-	-	0.231	0.016
GMFCS level II	-0.07	0.06	-0.20, 0.05		
GMFCS level III	-0.43	0.15	-0.73, -0.14		

CI: confidence interval; GMFCS: gross motor function classification system; SE: standard error

[a]bias-corrected and accelerated confidence interval

Supplemental Table 2. Linear regression analysis examining association between CHU-9D utility values (dependent variable) and GMFCS level.

CI: confidence interval; GMFCS: gross motor function classification system; SE: standard

	Coefficient	Bootstrap SE	95% CI[a]	R^2	p value
GMFCS level I	reference	-	-	0.071	0.170
GMFCS level II	0.02	0.03	-0.03, 0.07		
GMFCS level III	-0.07	0.05	-0.17, 0.02		

error

[a]bias-corrected and accelerated confidence interval

For peer review only

STROBE 2007 (v4) Statement—Checklist of items that should be included in reports of *cross-sectional studies*

Section/Topic	Item #	Recommendation	Reported on page #
**Title and abstract**	1	(*a*) Indicate the study's design with a commonly used term in the title or the abstract	2
		(*b*) Provide in the abstract an informative and balanced summary of what was done and what was found	2
**Introduction**			
Background/rationale	2	Explain the scientific background and rationale for the investigation being reported	4
Objectives	3	State specific objectives, including any prespecified hypotheses	5
**Methods**			
Study design	4	Present key elements of study design early in the paper	5
Setting	5	Describe the setting, locations, and relevant dates, including periods of recruitment, exposure, follow-up, and data collection	5
Participants	6	(*a*) Give the eligibility criteria, and the sources and methods of selection of participants	5
Variables	7	Clearly define all outcomes, exposures, predictors, potential confounders, and effect modifiers. Give diagnostic criteria, if applicable	5-6
Data sources/ measurement	8*	For each variable of interest, give sources of data and details of methods of assessment (measurement). Describe comparability of assessment methods if there is more than one group	5-6
Bias	9	Describe any efforts to address potential sources of bias	
Study size	10	Explain how the study size was arrived at	7
Quantitative variables	11	Explain how quantitative variables were handled in the analyses. If applicable, describe which groupings were chosen and why	6
Statistical methods	12	(*a*) Describe all statistical methods, including those used to control for confounding	6
		(*b*) Describe any methods used to examine subgroups and interactions	
		(*c*) Explain how missing data were addressed	
		(*d*) If applicable, describe analytical methods taking account of sampling strategy	
		(*e*) Describe any sensitivity analyses	
**Results**			

For peer review only

Participants	13*	(a) Report numbers of individuals at each stage of study—eg numbers potentially eligible, examined for eligibility, confirmed eligible, included in the study, completing follow-up, and analysed	7
		(b) Give reasons for non-participation at each stage	7
		(c) Consider use of a flow diagram	
Descriptive data	14*	(a) Give characteristics of study participants (eg demographic, clinical, social) and information on exposures and potential confounders	7
		(b) Indicate number of participants with missing data for each variable of interest	7
Outcome data	15*	Report numbers of outcome events or summary measures	7
Main results	16	(*a*) Give unadjusted estimates and, if applicable, confounder-adjusted estimates and their precision (eg, 95% confidence interval). Make clear which confounders were adjusted for and why they were included	Tables
		(*b*) Report category boundaries when continuous variables were categorized	
		(*c*) If relevant, consider translating estimates of relative risk into absolute risk for a meaningful time period	
Other analyses	17	Report other analyses done—eg analyses of subgroups and interactions, and sensitivity analyses	
**Discussion**			
Key results	18	Summarise key results with reference to study objectives	8
Limitations	19	Discuss limitations of the study, taking into account sources of potential bias or imprecision. Discuss both direction and magnitude of any potential bias	11
Interpretation	20	Give a cautious overall interpretation of results considering objectives, limitations, multiplicity of analyses, results from similar studies, and other relevant evidence	8
Generalisability	21	Discuss the generalisability (external validity) of the study results	11
**Other information**			
Funding	22	Give the source of funding and the role of the funders for the present study and, if applicable, for the original study on which the present article is based	12

*Give information separately for cases and controls in case-control studies and, if applicable, for exposed and unexposed groups in cohort and cross-sectional studies.

Note: An Explanation and Elaboration article discusses each checklist item and gives methodological background and published examples of transparent reporting. The STROBE checklist is best used in conjunction with this article (freely available on the Web sites of PLoS Medicine at http://www.plosmedicine.org/, Annals of Internal Medicine at http://www.annals.org/, and Epidemiology at http://www.epidem.com/). Information on the STROBE Initiative is available at www.strobe-statement.org.

For peer review only

BMJ Open

Comparison of the CHU-9D and the EQ-5D-Y instruments in children and young people with cerebral palsy; a cross-sectional study.

Journal:	*BMJ Open*
Manuscript ID	bmjopen-2020-037089.R2
Article Type:	Original research
Date Submitted by the Author:	26-Jul-2020
Complete List of Authors:	Ryan, Jennifer; Brunel University, College of Health and Life Sciences; Royal College of Surgeons in Ireland, Department of Public Health and Epidemiology<br>McKay, Ellen; Royal College of Surgeons in Ireland, Department of Public Health and Epidemiology<br>Anokye, Nana; Brunel University, Health Economics Research Group<br>Noorkoiv, Marika<br>Theis, Nicola; University of Gloucestershire, School of Sport and Exercise<br>Lavelle, Grace; King's College London, Institute of Psychiatry
**Primary Subject Heading**:	Health economics
Secondary Subject Heading:	Paediatrics, Neurology
Keywords:	HEALTH ECONOMICS, Developmental neurology & neurodisability < PAEDIATRICS, PAEDIATRICS

SCHOLARONE™
Manuscripts

BMJ

I, the Submitting Author has the right to grant and does grant on behalf of all authors of the Work (as defined in the below author licence), an exclusive licence and/or a non-exclusive licence for contributions from authors who are: i) UK Crown employees; ii) where BMJ has agreed a CC-BY licence shall apply, and/or iii) in accordance with the terms applicable for US Federal Government officers or employees acting as part of their official duties; on a worldwide, perpetual, irrevocable, royalty-free basis to BMJ Publishing Group Ltd ("BMJ") its licensees and where the relevant Journal is co-owned by BMJ to the co-owners of the Journal, to publish the Work in this journal and any other BMJ products and to exploit all rights, as set out in our licence.

The Submitting Author accepts and understands that any supply made under these terms is made by BMJ to the Submitting Author unless you are acting as an employee on behalf of your employer or a postgraduate student of an affiliated institution which is paying any applicable article publishing charge ("APC") for Open Access articles. Where the Submitting Author wishes to make the Work available on an Open Access basis (and intends to pay the relevant APC), the terms of reuse of such Open Access shall be governed by a Creative Commons licence – details of these licences and which Creative Commons licence will apply to this Work are set out in our licence referred to above.

Other than as permitted in any relevant BMJ Author's Self Archiving Policies, I confirm this Work has not been accepted for publication elsewhere, is not being considered for publication elsewhere and does not duplicate material already published. I confirm all authors consent to publication of this Work and authorise the granting of this licence.

For peer review only

Comparison of the CHU-9D and the EQ-5D-Y instruments in children and young people with cerebral palsy; a cross-sectional study.

Jennifer M. Ryan[1,2], Ellen McKay[2], Nana Anokye[1], Marika Noorkoiv[1], Nicola Theis[3], Grace Lavelle[4]

[1]College of Health and Life Sciences, Brunel University London, United Kingdom

[2]Department of Public Health and Epidemiology, RCSI, Ireland

[3]School of Sport and Exercise, University of Gloucestershire, Gloucester, United Kingdom

[4]Institute of Psychiatry, Psychology and Neuroscience, King's College London, United Kingdom

Corresponding author: Jennifer M. Ryan, College of Health and Life Sciences, Brunel University London, United Kingdom. Email: jennifer.ryan@brunel.ac.uk

Word count: 4295

For peer review only - http://bmjopen.bmj.com/site/about/guidelines.xhtml

Abstract

Objective: To compare the performance of the EuroQol 5D-youth (EQ-5D-Y) and child health utility 9D (CHU-9D) for assessing health-related quality of life (HRQoL) in children and young people (CYP) with cerebral palsy (CP).

Design: Cross-sectional study

Setting: England

Participants: Sixty-four CYP with cerebral palsy aged 10-19 years in Gross Motor Function Classification System (GMFCS) levels I-III.

Main outcome measures: Missing data were examined to assess feasibility. Associations between utility values and individual dimensions on each instrument were examined to assess convergent validity. Associations between utility values and GMFCS level were examined to assess known-group differences.

Results: Missing data were <5% for both instruments. Twenty participants (32.3%) and 11 participants (18.0%) reported full health for the EQ-5D-Y and CHU-9D, respectively. There was poor agreement between utilities from the two instruments (ICC=0.62; 95% limits of agreement -0.58 to 0.29). Correlations between EQ-5D-Y and CHU-9D dimensions were weak to moderate (r=0.25 to r=0.59). GMFCS level was associated with EQ-5D-Y utility values but not CHU-9D utility values.

Conclusions: The EQ-5D-Y and CHU-9D are feasible measures of HRQoL in CYP with CP. However, the two instruments demonstrate poor agreement and should not be used to measure and value HRQoL in CYP with CP interchangeably. We propose the CHU-9D may be preferable to use in this population as it assesses concepts that influence HRQoL among CYP with CP and provides less extreme utility values than the EQ-5D-Y.

Keywords: health economics; developmental neurology and neurodisability; paediatrics; cerebral palsy; EQ-5D-Y, CHU-9D

For peer review only

For peer review only - http://bmjopen.bmj.com/site/about/guidelines.xhtml

Strengths and limitations of this study

- This is the first study to compare the performance of the EQ-5D-Y and CHU-9D in a clinical population.
- Children and young people were recruited from the National Health Service, education and disability organisations across England.
- The findings are limited by a small sample and by inclusion of ambulatory individuals only.

For peer review only

For peer review only - http://bmjopen.bmj.com/site/about/guidelines.xhtml

Cerebral palsy (CP) is a heterogeneous disorder characterised by abnormal movement and posture. It is often co-existent with epilepsy, intellectual disability, and language, communication, or behavioural difficulties [1]. Its prevalence is 2 per 1,000 live births [2]; approximately 110,000 people live with CP in the UK [3]. Cerebral palsy is a lifelong condition. Management of CP encompasses medical, surgical and rehabilitation interventions [1]. While a large volume of research has examined the effectiveness of interventions for people with CP [4] there is limited research examining cost-effectiveness of such interventions.

Economic evaluation is used to inform the efficient allocation of resources in a healthcare setting. The cost utility analysis (CUA) is the type of economic evaluation recommended by the National Institute for Health and Care Excellence (NICE) particularly for interventions funded by NHS and personal social services [5]. The CUA commonly describes the relationship between costs and health benefits as the cost per quality-adjusted life year (QALY). QALYs incorporate both quantity and quality of life. QALYs are commonly calculated using an assessment of health-related quality of life (HRQoL) obtained from a preference-based instrument. Such instruments can provide a health state utility value, where 0 indicates a health state of equivalent value to being dead and 1.0 indicates full health, by applying a pre-specified algorithm based on preferences for health status identified in a specific population [6].

Generic HRQoL instruments are recommended for use in economic evaluations as they allow comparison across health care interventions and populations. However, if scores from generic measures differ, estimates of cost-effectiveness will be impacted, leading to uncertain conclusions regarding whether an intervention provides value for money. A number of generic measures have been used to obtain health state utility values from paediatric populations[7]. The most commonly used are the EuroQol 5D (EQ-5D), the Child Health Utility 9D (CHU-9D), the EuroQol 5D Youth version (EQ-5D-Y) and the Health Utilities Index 2 and 3 (HUI-2 and HUI-3) [7]. To date, however, only the HUI-2 and HUI-3 have been used to elicit utility values from children and young people (CYP) with CP and significant variation was reported in these [8].

The EQ-5D-Y and CHU-9D are two generic preference-based HRQoL instruments that were designed specifically for young people. The EQ-5D-Y is a youth modified version of the adult instrument, the EQ-5D, which was developed by revising the content and wording of the adult instrument [9]. The CHU-9D was developed from the outset for young people based on in-depth interviews with young people with chronic and acute health conditions [10]. Although the performance of the EQ-5D-Y and

For peer review only - http://bmjopen.bmj.com/site/about/guidelines.xhtml

CHU-9D has been examined in an adolescent population [11], their performance has not been examined among CYP with CP.

Given the financial cost of CP per annum is approximately AU$1.5 billion [12] and a wide range of interventions are currently available for people with CP [4], there is an increasing need for economic evaluation in this area. Prior to conducting economic evaluations, the performance of the EQ-5D-Y and CHU-9D in CYP with CP requires evaluation. The aim of this study was to compare the performance of the EQ-5D-Y and CHU-9D for assessing HRQoL in a community-based sample of CYP with CP. Specific objectives were to examine the feasibility of administering the instruments, to examine convergent validity, and to examine known-group differences for both measures.

Methods

Sample

CYP with CP who participated in a randomised controlled trial examining the effects of progressive resistance training were included in this study [13]. Data collected at baseline were used for this cross-sectional study. Participants were recruited from eight National Health Service (NHS) trusts in England, a special education needs school, a University and a primary care organisation in London, national organisations for people with disabilities, and by word of mouth. Inclusion criteria for participation in the trial were: aged 10-19 years with spastic CP and the ability to walk independently with or without a mobility aid (i.e., Gross Motor Function Classification System [GMFCS] levels I-III). Exclusion criteria for participation in the trial were: orthopaedic surgery of the lower limbs in the past 12 months, botulinum toxin type A injections or serial casting in the past 6 months, and insufficient cognition to comply with assessment procedures and the training programme. The study was approved by Brunel University London's College of Health and Life Sciences Research Ethics Committee and the Surrey Borders Research Ethics Committee (ref: 15/LO/0843). Participants 16 years and older provided written consent. Those under 16 years provided assent alongside written consent from a parent or guardian.

Data on the person's demographics, condition and HRQoL were collected using standardised questionnaires during an interview with a researcher at one time-point. Both HRQoL questionnaires were self-administered to all participants using the standardised instructions accompanying each measure. Assistance was provided by the researcher to read the questions if required. Further, the young person was allowed to ask their parent/guardian or researcher for assistance to answer the questions if required. Anatomical distribution was described as unilateral or bilateral [14]. Functional

For peer review only - http://bmjopen.bmj.com/site/about/guidelines.xhtml

mobility was classified according to the GMFCS. The GMFCS is a five-level classification system, where level I indicates most able and level V indicates most limited. Those in GMFCS level I are able to walk and run and climb stairs without assistance. Those in level II are able to walk in most settings but may use a hand-held mobility device indoors or wheeled mobility to travel long distances. Those in level III can walk using a hand-held mobility device but use a wheelchair or powered mobility outdoors. Participants selected a statement that best described their mobility based on descriptors of each GMFCS level [15]. Two physiotherapists retrospectively cross-referenced subjective ratings of GMFCS level against video recordings of participants, obtained as part of the baseline assessment.

Utility measurement

The EQ-5D-Y assesses a person's health across five dimensions. The five dimensions are "mobility", "looking after myself", "doing usual activities", "having pain or discomfort" and "feeling worried, sad or unhappy" [9]. Each dimension is rated on one of three levels (no problems, some problems, and a lot of problems) that describes a person's health today [9]. The EQ-5D-Y was developed by reviewing the applicability of the EQ-5D domain concepts and wording for children and adolescents [9]. The EQ-5D-Y is suitable for use in young people aged 8-19 years [16]. At present, there is no value set for the EQ-5D-Y. Although use of the adult value set (EQ-5D) for the EQ-5D-Y is not recommended, we calculated utilities using UK-based adult weights [17]. We acknowledge the limitation of this, but considered it to be the best method given the lack of weights for the EQ-5D-Y. Further, although the performance of the EQ-5D-Y using the adult value set has been examined in CYP with typical development [18], it has not been examined in a clinical population.

There are 9 dimension in the CHU-9D: "worried", "sad", "pain", "tired", "annoyed", "schoolwork", "sleep", "daily routine" and "ability to join in activities" [10]. For each dimension, the person describes how they are today according to one of five levels based on severity (e.g., not worried, a little bit worried, a bit worried, quite worried, very worried), which were determined from in-depth interviews with young people [10]. The CHU-9D is suitable for use in young people aged 7-17 years [10, 19]. The scoring algorithm based on the preferences of the UK adult general population was used to estimate utilities [20]. This method has been shown to be appropriate to use for young people [18].

Patient and Public Involvement

For peer review only

CYP with and without CP were involved in the design, conduct and dissemination plans of our research relating to the randomised controlled trial examining the effects of progressive resistance training.

Statistical analysis

The distribution of data was examined using histograms, Q-Q plots, and cross-tabulations. Mean and standard deviation (SD), median and interquartile range (IQR), frequencies and percentages were used to report the data as appropriate. We examined feasibility by reporting the number of participants with missing data and the percentage of missing data for each instrument. The instrument was considered feasible if missing data were <5% [21]. Participants who were missing data for an individual dimension of the EQ-5D-Y or CHU-9D were excluded from the calculation of utility values and from analyses involving that dimension.

To assess convergent validity, we calculated an intraclass correlation coefficient (ICC) between CHU-9D and EQ-5D-Y utility values. We interpreted an ICC >0.75 as indicating good agreement [22]. We compared mean utility between instruments using linear regression with a bootstrap procedure as there was evidence that residuals were not normally distributed. Bias corrected and accelerated bootstrap confidence intervals (CIs) were calculated from 2,000 replicates [23]. We also produced a Bland-Altman plot of the difference between the two instruments against their mean to examine agreement between utilities from the two instruments. We calculated 95% limits of agreement as mean difference±1.96SD [24]. We examined the association between 1) CHU-9D utility value and levels of each EQ-5D-Y dimension, and 2) EQ-5D-Y utility value and levels of each CHU-9D dimension, by calculating Spearman's correlation coefficients. We also examined associations between each dimension of the EQ-5D-Y and CHU-9D by calculating Spearman's correlation coefficients. Based on the description of each dimension and associations observed among adolescents with typical development [11], we hypothesised that the following dimensions would be correlated between the CHU-9D and EQ-5D-Y, respectively: "worried" vs. "feeling worried, sad or unhappy", "sad" vs. "feeling worried, sad or unhappy", "pain" vs "having pain/discomfort", "daily routine" vs. "doing usual activities", "daily routine" vs. "looking after myself", "able to join in" vs. "doing usual activities", and ""able to join in" vs. "mobility". To aid interpretation, we proposed a correlation of 0.10-0.39 to indicate a weak association, a correlation of 0.40-0.75 to indicate a moderate association, and a correlation of >0.75 to indicate a strong association [22, 25]. However, this interpretation should be used with caution given that cut-offs for interpreting correlation coefficients are arbitrary and may be inconsistent with other studies [25].

For peer review only - http://bmjopen.bmj.com/site/about/guidelines.xhtml

We examined known-group differences by fitting linear regression models using a bootstrap procedure to compare CHU-9D utility values and EQ-5D-Y utility values, respectively, across functional mobility as defined by the GMFCS. A bootstrap procedure was used as there was evidence that residuals were not normally distributed. For each model, utility value was the dependent variable and bias corrected and accelerated bootstrap CIs were calculated from 2,000 replicates [23]. We also compared the number of people experiencing no problems versus any problems for each CHU-9D and EQ-5D-Y dimension across GMFCS level, using a Chi^2 test. It was expected that CYP with better functional mobility would have higher utilities [26, 27]. MedCalc version 19.2.0 was used to produce the Bland-Altman plot. All other statistical analyses were performed using Stata version 13.

Results

Sixty-four participants were recruited to the study. One person did not complete the EQ-5D-Y or CHU-9D. Therefore, 63 participants were included in the analysis. Table 1 describes participant characteristics. The mean±SD age was 13.7±2.5 yr. The majority of participants (86%) were in GMFCS levels I or II indicating a mild lower limb impairment. The majority of participants were White British (59%) and attended a mainstream school (71%).

For the EQ-5D-Y, one participant did not provide a response to the “looking after myself” dimension. For the CHU-9D, two participants did not provide a response to the schoolwork dimension and one participant did not provide a response to the ability to “join in activities” dimension. Utility values for the EQ-5D-Y were therefore calculated for 62 out of 64 participants (96.9%) and utility values for the CHU-9D were calculated for 61 out of 64 participants (95.3%). Percentage missing data was 1.9% for the EQ-5D-Y and 2.0% for the CHU-9D.

The distribution of EQ-5D-Y and CHU-9D utilities is shown in Figures 1 and 2. For the EQ-5D-Y and CHU-9D, respectively, 20 participants (32.3%) and 11 participants (18.0%) reported full health. The median (IQR) EQ-5D-Y utility value was 0.80 (0.62 to 1.00). The mean±SD EQ-5D-Y utility value was 0.73±0.29 (range -0.17 to 1.00). Two participants (3.2%) reported a “worse than death” health state for the EQ-5D-Y. The median (IQR) CHU-9D utility value was 0.92 (0.83 to 0.96). The mean±SD CHU-9D utility value was 0.89±0.10 (range 0.56 to 1.00). There was poor agreement between utilities from the two instruments as indicated by an ICC of 0.62. The CHU-9D utility value was on average 0.15 (95% CI 0.09 to 0.25) higher than the EQ-5D-Y utility value. 95% limits of agreement were -0.58

For peer review only - http://bmjopen.bmj.com/site/about/guidelines.xhtml
For peer review only

to 0.29, indicating that the EQ-5D-Y utility value may be 0.29 higher or 0.58 lower than the CHU-9D utility value (Figure 3).

The percentages of reported problems across dimensions of the EQ-5D-Y and CHU-9D are presented in Tables 2 and 3. There was evidence of correlation between EQ-5D-Y utility values and level of severity for all dimensions of the CHU-9D except for the “sad” dimension. Correlations ranged from -0.12 to -0.64 (Table 2). For all dimensions of the EQ-5D-Y, the median CHU-9D utility value decreased with increasing levels of severity on the EQ-5D-Y dimensions (Table 3). For all dimensions of the EQ-5D-Y, the majority of respondents reported themselves in the least severe level (i.e. no problems). However, responses for most dimensions of the EQ-5D-Y were spread across all levels, from no problems to a lot of problems. The exception to this was the “worried, sad or unhappy” dimension; no participant reported feeling very worried, sad or unhappy. Similarly, for all dimensions of the CHU-9D except for “tired”, the majority of participants reported themselves in the least severe level. However, responses were not spread across all levels for each dimension. For the “worried”, “sad”, “pain”, “annoyed”, “sleep” and “daily routine” dimensions of the CHU-9D, no participant reported the most severe level.

Correlations between CHU-9D and EQ-5D-Y dimensions are presented in Table 4. Moderate correlations (r=0.43-0.59) were observed for all hypothesised associations, except for “worried” vs. “feeling worried, sad or unhappy” (r=0.25) and “daily routine” vs. “doing usual activities” (r=0.35). Several unexpected correlations were observed between dimensions. Namely, “schoolwork” on the CHU-9D was associated with “mobility” (r=0.56), “pain or discomfort” (r=0.55), and “doing usual activities” (r=0.44) on the EQ-5D-Y; “tired” on the CHU-9D was associated with “pain/discomfort” on the EQ-5D-Y (r = 0.40) and “annoyed” on the CHU-9D was associated with “worried/sad/unhappy” on the EQ-5D-Y (r = 0.53).

Median (IQR) utilities by GMFCS level are presented in Table 1. EQ-5D-Y utility value was associated with GMFCS level (R^2 = 0.231, p=0.016; supplemental Table 1). As expected, EQ-5D-Y utility value was on average 0.43 lower in individuals in level III compared to those in level I (95% CI 0.14 to 0.73) and 0.36 lower in individuals in level III compared to those in level II (95% CI 0.06 to 0.66). However, there was no difference in utility value between those in levels I and II. Although median CHU-9D utility value was lower in GMFCS level III compared to GMFCS level I and II (Table 1), there was no evidence that CHU-9D utility value was associated with GMFCS level (R^2 = 0.071, p=0.170; supplemental table 2). As presented in Table 5, the percentage of people reporting some or a lot of

For peer review only - http://bmjopen.bmj.com/site/about/guidelines.xhtml

problems on the “mobility” and “looking after myself” dimensions of the EQ-5D-Y differed according to GMFCS level, with the percentage of people reporting problems increasing from GMFCS levels I to III. The percentage of people reporting problems was not associated with GMFCS level for any other EQ-5D-Y dimension. There was also no evidence of an association between the percentage of people reporting problems and GMFCS level for any dimension of the CHU-9D (Table 6).

Discussion

The aim of this study was to compare the performance of the EQ-5D-Y and CHU-9D for assessing HRQoL in CYP with CP. Specific objectives were to examine feasibility, convergent validity, and known-group differences. To our knowledge, this is the first study to compare the EQ-5D-Y and CHU-9D in a clinical population. Although the results indicate that the two instruments are feasible to use when administered by interview to CYP with CP, the two instruments have poor agreement and may not be used interchangeably to measure and value HRQoL among CYP with CP. The EQ-5D-Y utility value was on average 0.15 lower than the CHU-9D utility value. However, there was considerable variation in individual differences between instruments. 95% limits of agreement demonstrated that the EQ-5D-Y utility value may be 0.29 higher or 0.58 lower than the CHU-9D utility value for an individual. Additionally, dimensions on each instrument that were hypothesised to measure similar concepts were only weakly to moderately associated.

Two studies compared the EQ-5D-Y and CHU-9D in CYP with typical development [11, 18]. Agreement between EQ-5D-Y and CHU-9D utility values was better among adolescents with typical development than among CYP with CP, with an ICC of 0.80 and much narrower 95% limits of agreement (-0.268 to 0.241) reported [11]. Although the median EQ-5D-Y utility value among CYP in this study was similar to that reported for Australian adolescents with typical development (0.80 versus 0.83), the median CHU-9D utility was higher (0.92 versus 0.83) [11]. The median CHU-9D utility reported by CYP with CP was more similar to that reported by children, aged 6-7 years, with typical development living in England (0.92 versus 0.90) and the median EQ-5D-Y utility was identical between these groups [18]. This may be because the study of children living in England used the same algorithms to estimate utilities as the current study, while the study of adolescents used algorithms developed in the Australian population. It is also plausible that CYP with CP have better HRQoL than CYP with typical development. A study of a large sample of CYP with CP across Europe found that CYP with CP had better QoL in five domains (moods and emotions, self-perception, autonomy, relationships with parents, school life) compared to CYP in the general population matched for age, sex and country [28].

For peer review only - http://bmjopen.bmj.com/site/about/guidelines.xhtml

Although the EQ-5D-Y utility value was on average lower than the CHU-9D utility value, 32% of participants reported full health when using the EQ-5D-Y compared to only 18% of participants when using the CHU-9D. A similar ceiling effect for the EQ-5D-Y was reported among CYP with typical development [11]. ~~Only 18% of CYP with CP reported full health when using the CHU-9D.~~ Although a large proportion of CYP had "perfect health" according to the EQ-5D-Y, two children reported a health state worse than death. Neither of these children reported the worst possible health state according to the CHU-9D. The CHU-9D may be more sensitive to varying severities of health because of the larger number of levels for each dimension. The CHU-9D is also the only generic measure that was specifically designed for CYP and the levels were determined from in-depth interviews with young people [10]. Further, as suggested previously, the extreme health states observed for the EQ-5D-Y may be a result of misapplication of the adult EQ-5D tariff to the health states defined by the EQ-5D-Y [18]. The findings from this study provide additional evidence that the EQ-5D-Y should not be used to measure utilities until a specific value set for the EQ-5D-Y is available.

Although we found associations between a number of dimensions on the EQ-5D-Y and CHU-9D as hypothesised, these associations were only weak to moderate, despite both instruments being administered at the same time-point, by the same researcher and in the same environment. These correlations were weaker than those reported among Australian adolescents with typical development [11]. In particular, the correlation between "feeling worried, sad or unhappy" on the EQ-5D-Y and "worried" on the CHU-9D was 0.70 among adolescents with typical development, compared to 0.25 among CYP with CP. This suggests that CYP with CP interpret these two dimensions differently and they do not measure the same concept in this population. The strongest correlation between EQ-5D-Y and CHU-9D dimensions among adolescents with typical development was between "having pain or discomfort" and "pain" (r=0.753) [11]. However, "having pain or discomfort" and "pain" were only moderately correlated in CYP with CP (r=0.47). Many CYP with CP experience frequent pain [29], and as a result, their interpretation of the EQ-5D-Y question about pain and discomfort may differ to their interpretation of the CHU-9D question about pain only. Indeed, fifty-six percent of CYP with CP reported no pain or discomfort on the EQ-5D-Y, while 62% reported no pain on the CHU-9D. Of the hypothesised correlations, only the correlation between "looking after myself" on the EQ-5D-Y and "daily routine" on the CHU-9D was stronger among CYP with CP compared to adolescents with typical development [11]. As the "looking after myself" dimension refers specifically to washing and dressing, this suggests that CYP with CP interpret self-care as part of their daily routine.

For peer review only - http://bmjopen.bmj.com/site/about/guidelines.xhtml

We also observed a number of unexpected associations between EQ-5D-Y and CHU-9D dimensions. "Schoolwork" on the CHU-9D was associated with "mobility", "having pain or discomfort" and "doing usual activities" on the EQ-5D-Y. Approximately 45% of CYP with CP have an intellectual disability, which may range from mild to severe [30]. Although we excluded individuals with insufficient cognition to comply with assessment procedures, some participants may have had a mild intellectual disability. As CYP with intellectual disability are likely to have more severely impaired physical functioning [30], it is possible that CYP with intellectual disability have more problems completing schoolwork and more problems with mobility and doing usual activities. Alternatively, CYP with more severe physical impairment, who will have more problems with mobility and doing usual activities, may also have more problems with completing schoolwork as a result of their physical impairment.

We observed an association between EQ-5D-Y utility values and GMFCS level but not between CHU-9D utility values and GMFCS level. Two studies reported that utility values obtained from the HUI-3 differed according to GMFCS level [26, 27]. However, regardless of the instrument used, we found that those in GMFCS levels I and II have a similar mean utility value, while those in GMFCS level III have a lower mean utility value compared to levels I and II. The lack of statistical evidence of an association between CHU-9D utility value and GMFCS level may be due to the narrower utility range of the CHU-9D, which resulted in smaller incremental changes between levels. There was also a small number of participants in GMFCS level III, which likely resulted in reduced statistical power to detect differences between groups. However, it is also plausible that HRQoL is not associated with functional mobility. When condition-specific measures of QoL were used, associations between QoL and GMFCS level were not observed [27, 28].

The mean EQ-5D-Y utility value for each GMFCS level was similar to values obtained from the HUI-3 in one study (0.84, 0.50 and 0.39 for adolescents in GMFCS levels I, II and III, respectively) [27] but not similar to those in a second study (0.67, 0.59 and 0.43 in GMFCS levels I, II and III, respectively) [26]. The mean CHU-9D utility values for each GMFCS level were higher than those obtained from the HUI-3 [26, 27]. Discrepancies may be due to differences in the algorithms used to derive utility values. HUI-3 utility values were derived in one study using preferences from the general adult population in Canada [27]. The second study did not state the preferences used to derive HUI-3 utility values [26]. Differences in utilities between the CHU-9D and HUI-3 do not necessarily indicate that the CHU-9D is inaccurate. The HUI-3 was only weakly correlated with a condition-specific

For peer review only - http://bmjopen.bmj.com/site/about/guidelines.xhtml

measure of HRQoL in CYP with CP [27]. This weak correlation highlights that condition-specific measures and generic measures of HRQoL may not assess similar concepts. However, unlike generic measures, condition-specific measures are not recommended for evaluations across different conditions [31]. Although a generic measure, the CHU-9D may be more likely than the EQ-5D-Y and HUI to capture dimensions of HRQoL that are important to CYP with CP. In particular, the CHU-9D includes more questions about psychological difficulties and pain, which are associated with QoL among CYP with CP [28].

Limitations

The use of adult weights to value EQ-5D-Y health states is a limitation of this study. It has previously been demonstrated that values for health states ascribed by adults differ to corresponding health states ascribed by children [32, 33]. However, we used adult weights in the absence of value sets for the EQ-5D-Y. The findings of this study are limited by a small sample. In particular there were a small number of participants in GMFCS level III. Although the sample included CYP with CP in GMFCS levels I-III, which represents about 70% of individuals with CP [34], the findings may not be applicable to non-ambulatory CYP. As the sample volunteered to participate, they may have a higher HRQoL than those who did not volunteer.

In conclusion, the findings of this study illustrate that the EQ-5D-Y and the CHU-9D are feasible to use among CYP with CP. However, there is poor agreement between utility values elicited from the two instruments and they should not be used interchangeably to measure and value HRQoL in CYP with CP. This study provides further evidence that it is not appropriate to use the adult EQ-5D tariff to derive utility values from the EQ-5D-Y. Additionally, we propose that the CHU-9D is preferable to the EQ-5D-Y for measuring HRQoL among CYP with CP because it was developed based on interviews with CYP, it assesses concepts that influence QoL among CYP with CP, and produces less extreme values than the EQ-5D-Y. However, this study is limited by a small sample size and more research is needed to compare these instruments in CYP with CP and in other clinical populations.

Competing interests: None declared.

Author statement: JR and GL conceived the study. JR, GL and NA designed the study. GL, JR, MN and NT acquired data. JR, GL and EM performed the analysis. NA contributed to analysis and interpretation of the data. JR, GL and EM drafted the manuscript. NA, MN and NT critically revised the manuscript. All authors approved the final manuscript.

Data sharing statement: Data are available upon reasonable request.

For peer review only - http://bmjopen.bmj.com/site/about/guidelines.xhtml

Ethics approval: The study was approved by Brunel University London's College of Health and Life Sciences Research Ethics Committee and the Surrey Borders Research Ethics Committee (ref: 15/LO/0843).

Funding: Action Medical Research and the Chartered Society of Physiotherapy Charitable Trust have jointly funded this project, and it is supported by a generous grant from The Henry Smith Charity (GN2340).

References

1. Colver A, Fairhurst C, Pharoah PO. Cerebral palsy. *Lancet* 2014;383:1240-9.
2. Oskoui M, Coutinho F, Dykeman J, *et al.* An update on the prevalance of cerebral palsy: a systematic review and meta-analysis. *Dev Med Child Neurol* 2013;55:509-19.
3. Department of Health Long-term Conditions National Service Framework Team. The National Service Framework for Long-Term Conditions. *Department of Health* 2005.
4. Novak I, McIntyre S, Morgan C, *et al.* A systematic review of interventions for children with cerebral palsy: state of the evidence. *Dev Med Child Neurol* 2013;55:885-910.
5. NICE. Developing NICE guidelines: the manual. London; 2018.
6. Whitehead SJ, Ali S. Health outcomes in economic evaluation: the QALY and utilities. *Br Med Bull* 2010;96:5-21.
7. Thorrington D, Eames K. Measuring Health Utilities in Children and Adolescents: A Systematic Review of the Literature. *PloS One,* 2015;10:e0135672.
8. Tonmukayakul U, Le LK, Mudiyanselage SB, *et al.* A systematic review of utility values in children with cerebral palsy. *Qual Life Res* 2019;28:1-12.
9. Wille N, Badia X, Bonsel G, *et al.* Development of the EQ-5D-Y: a child-friendly version of the EQ-5D. *Qual Life Res* 2010;19:875-86.
10. Stevens K. Developing a descriptive system for a new preference-based measure of health-related quality of life for children. *Qual Life Res* 2009;18:1105-13.
11. Chen G, Flynn T, Stevens K, *et al.* Assessing the Health-Related Quality of Life of Australian Adolescents: An Empirical Comparison of the Child Health Utility 9D and EQ-5D-Y Instruments. *Value Health* 2015;18:432-8.
12. Access Economics. The economic impact of cerebral palsy in Australia in 2007. Canberra, Australia: Access Economics 2008.
13. Ryan JM, Theis N, Kilbride C, *et al.* Strength Training for Adolescents with cerebral palsy (STAR): study protocol of a randomised controlled trial to determine the feasibility, acceptability and efficacy of resistance training for adolescents with cerebral palsy. *BMJ open.* 2016;6:e012839.
14. Rosenbaum P, Paneth N, Leviton A, *et al.* A report: the definition and classification of cerebral palsy April 2006. *Dev Med Child Neurol Suppl* 2007;109:8-14.
15. Palisano R, Rosenbaum P, Walter S, *et al.* Development and reliability of a system to classify gross motor function in children with cerebral palsy. *Dev Med Child Neurol* 1997;39:214-23.
16. Ravens-Sieberer U, Wille N, Badia X, *et al.* Feasibility, reliability, and validity of the EQ-5D-Y: results from a multinational study. *Qual Life Res* 2010;19:887-97.
17. Dolan P. Modelling valuations for EuroQol health states. *Med Care* 1997;35:1095-108.
18. Canaway AG, Frew EJ Measuring preference-based quality of life in children aged 6-7 years: a comparison of the performance of the CHU-9D and EQ-5D-Y--the WAVES pilot study. *Qual Life Res* 2013;22:173-83.
19. Ratcliffe J, Stevens K, Flynn T, *et al.* An assessment of the construct validity of the CHU9D in the Australian adolescent general population. *Qual Life Res* 2012;21:717-25.
20. Stevens K. Valuation of the Child Health Utility 9D Index. *Pharmacoeconomics* 2012;30:729-747.

For peer review only - http://bmjopen.bmj.com/site/about/guidelines.xhtml

21. Little TD, Jorgensen TD, Lang KM, Whitney E, Moore G. On the Joys of Missing Data. *J Pediatr Psychol* 2014;39:151–62.
22. Fleiss JL, Cohen J. The equivalence of weighted kappa and the intraclass correlation coefficient as measures of reliability. *Educ Psychol Meas* 1973;33:613–9.
23. Good P. Permutation, Parametric, and Bootstrap Tests of Hypotheses. Third edition. 2005 Springer.
24. Bland JM, Altman DG. Statistical methods for assessing agreement between two methods of clinical measurement. *Lancet* 1986;1;307-10.
25. Schober P, Boer C, Schwarte LA. Correlation Coefficients: Appropriate Use and Interpretation. *Anesth Analg*. 2018;126:1763-8.
26. Young NL, Rochon TG, McCormick A, *et al.* The health and quality of life outcomes among youth and young adults with cerebral palsy. *Arch Phys Med Rehabil* 2010;91:143-8.
27. Rosenbaum PL, Livingston MH, Palisano RJ, *et al.* Quality of life and health-related quality of life of adolescents with cerebral palsy. *Dev Med Child Neurol* 2007;49:516-21.
28. Colver A, Rapp M, Eisemann N, *et al.* Self-reported quality of life of adolescents with cerebral palsy: a cross-sectional and longitudinal analysis. *Lancet* 2015;385:705-16.
29. Ostojic K, Paget S, Kyriagis M, Morrow A. Acute and Chronic Pain in Children and Adolescents With Cerebral Palsy: Prevalence, Interference, and Management. *Arch Phys Med Rehabil* 2020;101:213-9.
30. Reid SM, Meehan EM, Arnup SJ, *et al.* Intellectual disability in cerebral palsy: a population-based retrospective study. *Dev Med Child Neurol* 2018;60:687-94.
31. Seow LSE, Tan THG, Abdin E, *et al.* Comparing disease-specific and generic quality of life measures in patients with schizophrenia. *Psychiatry Res* 2019;273:387-93.
32.Kind P, Klose K, Gusi N, Olivares PR, Greiner W. Can adult weights be used to value child health states? Testing the influence of perspective in valuing EQ-5D-Y. *Qual Life Res* 2015;24:2519-39.
33. Kreimeier S, Oppe M, Ramos-Goñi JM, Cole A, Devlin N, Herdman M, *et al*. Valuation of EuroQol five-dimensional questionnaire, youth version (EQ-5D-Y) and EuroQol five-dimensional questionnaire, three-level version (EQ-5D-3L) health states: the impact of wording and perspective. *Value Health* 2018;21:1291-8.
34. Children and young people with cerebral palsy in Northern Ireland (1981-2008). A comprehensive report from the Northern Ireland Cerebral Palsy Register. HSC Public Health Agency and Queen's University Belfast.

For peer review only - http://bmjopen.bmj.com/site/about/guidelines.xhtml

Table 1. Participant characteristics

	n (%)	Mean (SD)	Range	Mean (SD) EQ-5D-Y utility value	Median (IQR) EQ-5D-Y utility value	Mean (SD) CHU-9D utility value	Median (IQR) CHU-9D utility value
Age, yr	63	13.7 (2.5)	10-19	-	-	-	-
Female	26 (41.3)			0.77 (0.29)	0.83 (0.69-1.00)	0.90 (0.08)	0.92 (0.83-0.97)
Male	37 (58.7)			0.71 (0.29)	0.77 (0.62-0.93)	0.88 (0.11)	0.92 (0.81-0.95)
Height, cm	63	154.3 (12.7)	131.5-180.9	-	-	-	-
Mass, kg	63	49.3 (13.7)	27.4-78.5	-	-	-	-
**Ethnicity**							
White British	37 (58.7)			0.80 (0.20)	0.81 (0.69-1.00)	0.89 (0.09)	0.92 (0.84-0.95)
Black or Black British	5 (7.9)			0.62 (0.44)	0.81 (0.19-1.00)	0.90 (0.10)	0.90 (0.81-1.00)
Asian or Asian British	9 (14.3)			0.59 (0.49)	0.73 (0.20-1.00)	0.88 (0.12)	0.92 (0.89-0.95)
Other	12 (19.0)			0.69 (0.22)	0.75 (0.57-0.81)	0.87 (0.13)	0.87 (0.82-0.97)
**GMFCS level**							
I	29 (46.0)			0.82 (0.20)	0.81 (0.73-1.00)	0.89 (0.11)	0.92 (0.82-0.95)
II	25 (39.7)			0.75 (0.25)	0.75 (0.62-1.00)	0.90 (0.08)	0.92 (0.86-0.97)
III	9 (14.3)			0.39 (0.43)	0.44 (0.00-0.76)	0.82 (0.12)	0.83 (0.72-0.89)
**Distribution**							
Unilateral	31 (49.2)			0.81 (0.18)	0.80 (0.69-1.00)	0.91 (0.09)	0.92 (0.87-0.98)
Bilateral	32 (50.8)			0.66 (0.35)	0.75 (0.59-1.00)	0.87 (0.11)	0.87 (0.81-0.95)
**Type of School**							

For peer review only

For peer review only - http://bmjopen.bmj.com/site/about/guidelines.xhtml

Mainstream school	45 (71.4)	0.77 (0.24)	0.80 (0.67-1.00)	0.88 (0.08)	0.90 (0.82-0.95)
SEN	13 (20.6)	0.62 (0.41)	0.80 (0.44-0.85)	0.89 (0.11)	0.92 (0.86-0.98)
Further college education	2 (3.2)	0.85 (0.22)	0.85 (0.69-1.00)	0.99 (0.01)	0.99 (0.98-1.00)
University	3 (4.8)	0.67 (0.45)	0.85 (0.16-1.00)	0.85 (0.25)	1.00 (0.56-1.00)

SD: standard deviation; GMFM: Gross motor function classification system; SEN: Special Needs Education

For peer review only

Table 2. Number and percentage of participants reporting problems across CHU-9D dimensions

CHU-9D dimensions	levels	Frequency (%)	n	Median±IQR EQ-5D-Y utility value	rho	p value
Worried	Not worried	82.5	52	0.81±0.29	-0.38	0.002
	A little bit	15.9	10	0.55±0.66		
	A bit	1.6	1	-0.11		
	Quite	0	0	-		
	Very	0	0	-		
Sad	Not sad	93.7	59	0.80±0.34	-0.12	0.350
	A little bit	3.2	2	0.50±0.69		
	A bit	3.2	2	0.68±0.33		
	Quite	0	0	-		
	Very	0	0	-		
Pain	No pain	61.9	39	0.85±0.29	-0.42	<0.001
	A little bit	30.2	19	0.73±0.23		
	A bit	3.2	2	0.39±0.60		
	Quite	4.8	3	0.16±0.73		
	A lot	0	0	-		
Tired	Not tired	36.5	23	0.82±0.27	-0.44	<0.001
	A little bit	42.9	27	0.81±0.31		
	A bit	9.5	6	0.59±0.07		
	Quite	7.9	5	0.19±0.46		
	Very	3.2	2	0.45±0.52		
Annoyed	Not annoyed	84.1	53	0.81±0.30	-0.27	0.034
	A little bit	11.1	7	0.62±0.33		
	A bit	3.2	2	0.50±0.69		
	Quite	1.6	1	0.52		
	Very	0	0	-		

For peer review only

For peer review only - http://bmjopen.bmj.com/site/about/guidelines.xhtml

Schoolwork/homework	No problems	63.5	40	0.93±0.21	-0.64	<0.001
	Few problems	17.5	11	0.68±0.10		
	Some problems	14.3	9	0.55±0.52		
	Many problems	0	0	-		
	Can't do schoolwork	1.6	1	0.20		
	Missing	3.2	2	-		
Sleep	No problems	71.4	45	0.81±0.29	-0.38	0.002
	Few problems	20.6	13	0.70±0.24		
	Some problems	4.8	3	0.73±0.07		
	Many problems	3.2	2	0.02±0.27		
	Can't sleep	0	0	-		
Daily routine	No problems	74.6	47	0.81±0.27	-0.48	<0.001
	Few problems	15.9	10	0.52±0.59		
	Some problems	7.9	5	0.66±0.14		
	Many problems	1.6	1	0.09		
	Can't do daily routine	0	0	-		
Able to join in activities	Any activities	57.1	36	0.85±0.27	-0.43	<0.001
	Most activities	27.0	17	0.73±0.23		
	Some activities	7.9	5	0.60±0.33		
	Few activities	4.8	3	0.71±0.33		
	No activities	1.6	1	-0.11		
	Missing	1.6	1	-		

For peer review only - http://bmjopen.bmj.com/site/about/guidelines.xhtml

Table 3. Number and percentage of participants reporting problems across EQ-5D-Y dimensions

EQ-5D-Y dimensions	levels	Frequency (%)	n	Median±IQR CHU-9D utility value	rho	p value
Mobility	No	50.8	32	0.95±0.09	-0.52	<0.001
	Some	44.4	28	0.84±0.13		
	A lot of	4.8	3	0.75±0.05		
Looking after myself	No	66.7	42	0.94±0.09	-0.46	<0.001
	Some	27.0	17	0.83±0.10		
	A lot of	4.8	3	0.72±0.27		
	Missing	1.6	1	0.78		
Usual activities	No	66.7	42	0.93±0.11	-0.54	<0.001
	Some	28.6	18	0.83±0.15		
	A lot of	4.8	3	0.72±0.16		
Pain or discomfort	No	55.6	35	0.95±0.11	-0.54	<0.001
	Some	38.1	24	0.86±0.16		
	A lot of	6.3	4	0.72±0.24		
Worried, sad or unhappy	Not	84.1	53	0.92±0.11	-0.44	<0.001
	A bit	15.9	10	0.79±0.05		
	Very	0	0	-		

For peer review only

Table 4. Spearman rank correlation coefficients between CHU-9D and EQ-5D-Y dimensions.

**EQ-5D-Y dimensions**		**CHU-9D dimensions**								
		**Worried**	**Sad**	**Pain**	**Tired**	**Annoyed**	**Schoolwork**	**Sleep**	**Daily routine**	**Activities**
**Mobility**	*r*	***0.30***	*-0.01*	***0.35***	***0.36***	*0.19*	***0.56***[a]	***0.31***	*0.19*	***0.49***[b]
	p	**0.017**	0.950	**0.006**	**0.004**	0.140	**<0.001**	**0.013**	0.144	**<0.001**
**Looking after myself**	*r*	***0.26***[b]	*-0.04*[b]	*0.21*[b]	*0.22*[b]	*0.08*[b]	***0.39***[c]	***0.27***[b]	***0.59***[b]	***0.36***[a]
	p	**0.040**	0.741	0.098	0.086	0.525	**0.002**	**0.031**	**<0.001**	**0.004**
**Activities**	*r*	***0.36***	*0.08*	***0.30***	*0.23*	***0.25***	***0.44***[a]	***0.38***	***0.35***	***0.54***[b]
	p	**0.004**	0.543	**0.017**	0.065	**0.049**	**<0.001**	**0.002**	**0.006**	**<0.001**
**Pain or discomfort**	*r*	***0.33***	*0.07*	***0.47***	***0.40***	*0.22*	***0.55***[a]	***0.39***	***0.25***	***0.32***[b]
	p	**0.008**	0.600	**<0.001**	**0.001**	0.090	**<0.001**	**0.002**	**0.048**	**0.011**
**Worried, sad, unhappy**	*r*	***0.25***	***0.43***	*0.19*	***0.26***	***0.53***	***0.39***[a]	*-0.01*	***0.33***	*0.15*[b]
	p	**0.048**	**<0.001**	0.143	**0.039**	**<0.001**	**0.002**	0.944	**0.008**	0.239

Note: correlations with p value <0.05 highlighted in bold; n=63 for all analyses unless stated otherwise.
[a]n=61; [b]n=62; [c]n=60

For peer review only

Table 5. Percentage reporting problems on each EQ-5D-Y dimension across GMFCS level

EQ-5D-Y dimensions	levels	GMFCS level I	GMFCS level II	GMFCS level III	*p value*[a]
		n=29	n=25	n=9	
Mobility	No	69%	44%	11%	0.007
	Some	31%	56%	56%	
	A lot of	0%	0%	33%	
Looking after myself	No	83%	56%	50%	0.036
	Some	17%	40%	25%	
	A lot of	0%	4%	25%	
Usual activities	No	72%	72%	33%	0.072
	Some	28%	24%	44%	
	A lot of	0%	4%	22%	
Pain or discomfort	No	59%	60%	33%	0.348
	Some	38%	36%	44%	
	A lot of	3%	4%	22%	
Worried, sad or unhappy	No	79%	92%	78%	0.380
	Some	21%	8%	22%	
	A lot of	0%	0%	0%	

[a]Comparing number of people experiencing “no” problems versus any problems (i.e., some and a lot of) across GMFCS levels

For peer review only

Table 6. Percentage reporting problems on each CHU-9D dimension across GMFCS level

CHU-9D dimensions	levels	GMFCS level I	GMFCS level II	GMFCS level III	*p value*[a]
		n=29	n=25	n=9	
Worried	Not worried	83%	84%	78%	0.914
	A little bit	17%	16%	11%	
	A bit	0%	0%	11%	
	Quite	0%	0%	0%	
	Very	0%	0%	0%	
Sad	Not sad	86%	100%	100%	0.082
	A little bit	7%	0%	0%	
	A bit	7%	0%	0%	
	Quite	0%	0%	0%	
	Very	0%	0%	0%	
Pain	No pain	55%	72%	56%	0.408
	A little bit	38%	24%	22%	
	A bit	0%	4%	11%	
	Quite	7%	0%	11%	
	A lot	0%	0%	0%	
Tired	Not tired	31%	44%	33%	0.601
	A little bit	52%	32%	44%	
	A bit	7%	12%	11%	
	Quite	10%	8%	0%	

For peer review only

For peer review only - http://bmjopen.bmj.com/site/about/guidelines.xhtml

	Very	0%	4%	11%	
Annoyed	Not annoyed	72%	96%	89%	0.056
	A little bit	17%	4%	11%	
	A bit	7%	0%	0%	
	Quite	4%	0%	0%	
	Very	0%	0%	0%	
Schoolwork/homework	No problems	72%	64%	43%	0.104
	Few problems	17%	16%	29%	
	Some problems	10%	20%	14%	
	Many problems	0%	0%	14%	
	Can't do schoolwork	0%	0%	0%	
Sleep	Not	79%	68%	56%	0.343
	A little bit	10%	28%	33%	
	A bit	7%	4%	0%	
	Quite	4%	0%	11%	
	Very	0%	0%	0%	
Daily routine	Not	83%	72%	56%	0.243
	A little bit	10%	20%	22%	
	A bit	7%	4%	22%	
	Quite	0%	4%	0%	
	Very	0%	0%	0%	
Able to join in activities	Not	69%	52%	38%	0.135
	A little bit	24%	28%	38%	

For peer review only

A bit	3.5%	12%	13%
Quite	3.5%	8%	0%
Very	0%	0%	13%

[a]Comparing number of people experiencing “no” problems versus any problems (i.e., a little bit, a bit, quite and very) across GMFCS levels

For peer review only

For peer review only - http://bmjopen.bmj.com/site/about/guidelines.xhtml

Figure legend

Figure 1. Distribution of utility values for the EQ-5D-Y.

Figure 2. Distribution of utility values for the CHU-9D.

Figure 3. Bland-Altman plot of differences between EQ-5D-Y and CHU-9D utility values against average of EQ-5D-Y and CHU-9D utility values.

For peer review only

For peer review only - http://bmjopen.bmj.com/site/about/guidelines.xhtml

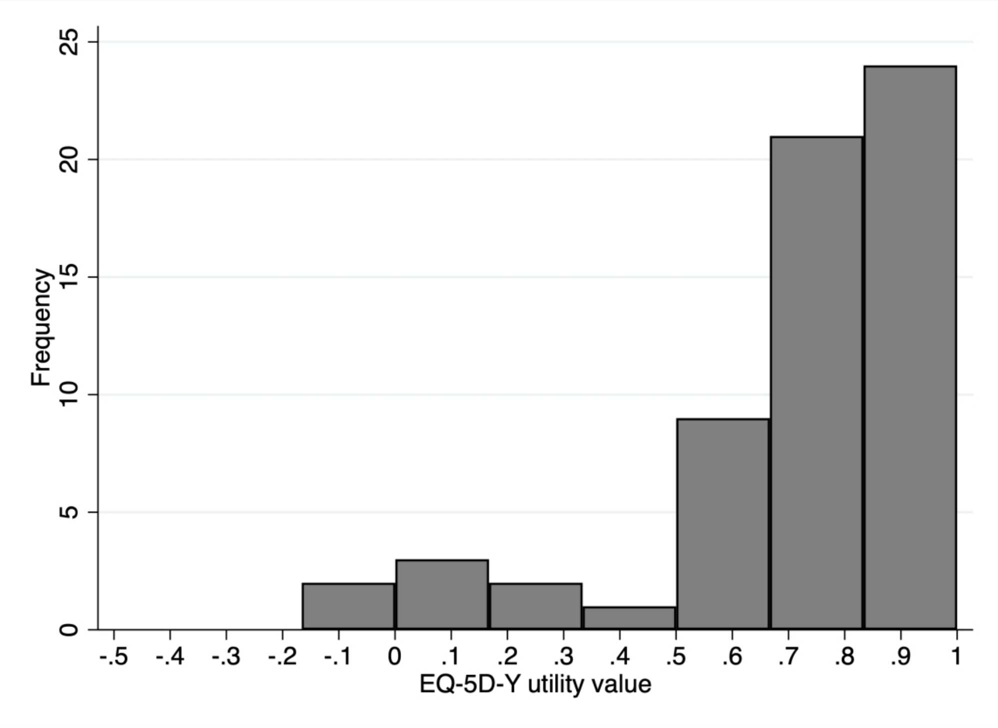

Figure 1. Distribution of utility values for the EQ-5D-Y.

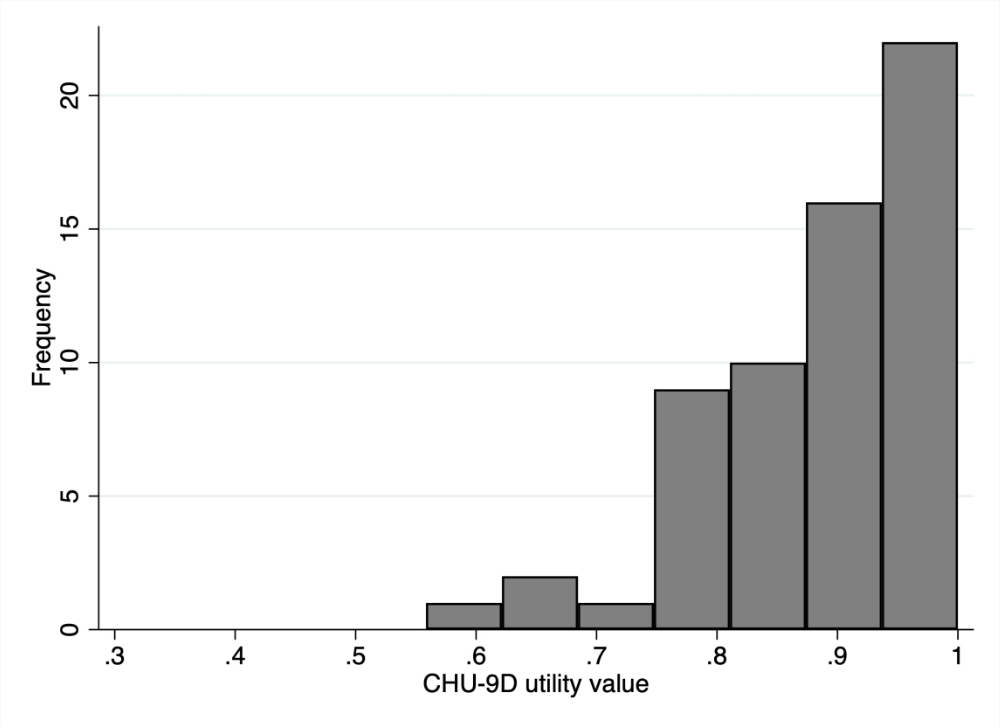

Figure 2. Distribution of utility values for the CHU-9D.

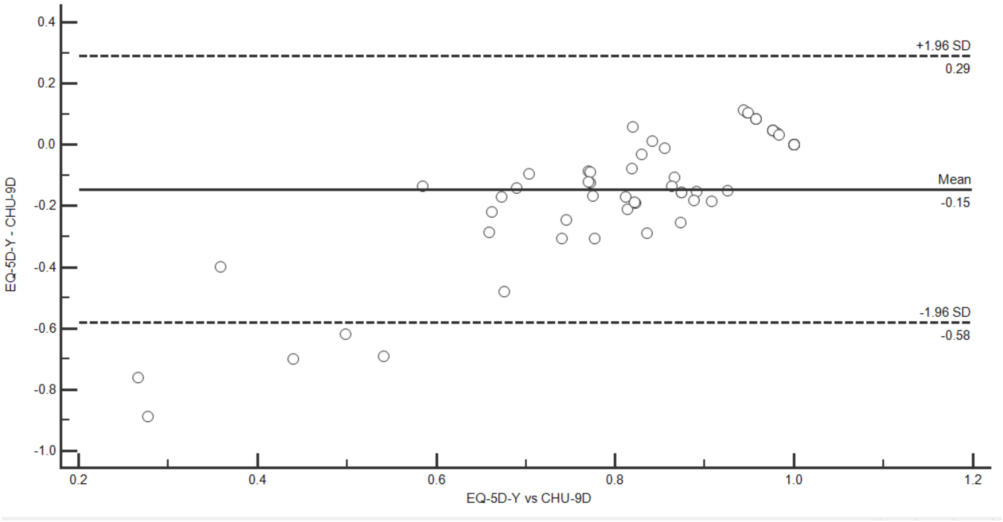

Figure 3. Bland-Altman plot of differences between EQ-5D-Y and CHU-9D utility values against average of EQ-5D-Y and CHU-9D utility values.

Supplemental Table 1. Linear regression analysis examining association between EQ-5D-Y utility values (dependent variable) and GMFCS level.

	Coefficient	Bootstrap SE	95% CI[a]	R^2	p value
GMFCS level I	reference	-	-	0.231	0.016
GMFCS level II	-0.07	0.06	-0.20, 0.05		
GMFCS level III	-0.43	0.15	-0.73, -0.14		

CI: confidence interval; GMFCS: gross motor function classification system; SE: standard error

[a]bias-corrected and accelerated confidence interval

For peer review only

Supplemental Table 2. Linear regression analysis examining association between CHU-9D utility values (dependent variable) and GMFCS level.

CI: confidence interval; GMFCS: gross motor function classification system; SE: standard

	Coefficient	Bootstrap SE	95% CI[a]	R^2	p value
GMFCS level I	reference	-	-	0.071	0.170
GMFCS level II	0.02	0.03	-0.03, 0.07		
GMFCS level III	-0.07	0.05	-0.17, 0.02		

error

[a]bias-corrected and accelerated confidence interval

STROBE 2007 (v4) Statement—Checklist of items that should be included in reports of *cross-sectional studies*

Section/Topic	Item #	Recommendation	Reported on page #
**Title and abstract**	1	(*a*) Indicate the study's design with a commonly used term in the title or the abstract	2
		(*b*) Provide in the abstract an informative and balanced summary of what was done and what was found	2
**Introduction**			
Background/rationale	2	Explain the scientific background and rationale for the investigation being reported	4
Objectives	3	State specific objectives, including any prespecified hypotheses	5
**Methods**			
Study design	4	Present key elements of study design early in the paper	5
Setting	5	Describe the setting, locations, and relevant dates, including periods of recruitment, exposure, follow-up, and data collection	5
Participants	6	(*a*) Give the eligibility criteria, and the sources and methods of selection of participants	5
Variables	7	Clearly define all outcomes, exposures, predictors, potential confounders, and effect modifiers. Give diagnostic criteria, if applicable	5-6
Data sources/ measurement	8*	For each variable of interest, give sources of data and details of methods of assessment (measurement). Describe comparability of assessment methods if there is more than one group	5-6
Bias	9	Describe any efforts to address potential sources of bias	
Study size	10	Explain how the study size was arrived at	7
Quantitative variables	11	Explain how quantitative variables were handled in the analyses. If applicable, describe which groupings were chosen and why	6
Statistical methods	12	(*a*) Describe all statistical methods, including those used to control for confounding	6
		(*b*) Describe any methods used to examine subgroups and interactions	
		(*c*) Explain how missing data were addressed	
		(*d*) If applicable, describe analytical methods taking account of sampling strategy	
		(*e*) Describe any sensitivity analyses	
**Results**			

For peer review only

Participants	13*	(a) Report numbers of individuals at each stage of study—eg numbers potentially eligible, examined for eligibility, confirmed eligible, included in the study, completing follow-up, and analysed	7
		(b) Give reasons for non-participation at each stage	7
		(c) Consider use of a flow diagram	
Descriptive data	14*	(a) Give characteristics of study participants (eg demographic, clinical, social) and information on exposures and potential confounders	7
		(b) Indicate number of participants with missing data for each variable of interest	7
Outcome data	15*	Report numbers of outcome events or summary measures	7
Main results	16	(*a*) Give unadjusted estimates and, if applicable, confounder-adjusted estimates and their precision (eg, 95% confidence interval). Make clear which confounders were adjusted for and why they were included	Tables
		(*b*) Report category boundaries when continuous variables were categorized	
		(*c*) If relevant, consider translating estimates of relative risk into absolute risk for a meaningful time period	
Other analyses	17	Report other analyses done—eg analyses of subgroups and interactions, and sensitivity analyses	
**Discussion**			
Key results	18	Summarise key results with reference to study objectives	8
Limitations	19	Discuss limitations of the study, taking into account sources of potential bias or imprecision. Discuss both direction and magnitude of any potential bias	11
Interpretation	20	Give a cautious overall interpretation of results considering objectives, limitations, multiplicity of analyses, results from similar studies, and other relevant evidence	8
Generalisability	21	Discuss the generalisability (external validity) of the study results	11
**Other information**			
Funding	22	Give the source of funding and the role of the funders for the present study and, if applicable, for the original study on which the present article is based	12

*Give information separately for cases and controls in case-control studies and, if applicable, for exposed and unexposed groups in cohort and cross-sectional studies.

Note: An Explanation and Elaboration article discusses each checklist item and gives methodological background and published examples of transparent reporting. The STROBE checklist is best used in conjunction with this article (freely available on the Web sites of PLoS Medicine at http://www.plosmedicine.org/, Annals of Internal Medicine at http://www.annals.org/, and Epidemiology at http://www.epidem.com/). Information on the STROBE Initiative is available at www.strobe-statement.org.

For peer review only
